# Recent European drying and its link to prevailing large-scale atmospheric patterns

**DOI:** 10.1038/s41598-023-48861-4

**Published:** 2023-12-08

**Authors:** Sigrid J. Bakke, Monica Ionita, Lena M. Tallaksen

**Affiliations:** 1 Department of Geosciences, University of Oslo, Oslo, Norway; 2 Alfred Wegener Institute, Helmholtz Centre for Polar and Marine Research, Bremerhaven, Germany; 3 Faculty of Forestry, “Stefan cel Mare” University of Suceava, Suceava, Romania; 4 Present Address: The Norwegian Water Resources and Energy Directorate, Oslo, Norway

**Keywords:** Climate sciences, Hydrology, Natural hazards

## Abstract

The extreme 2018 and 2022 droughts pose as recent examples of a series of drought events that have hit Europe over the last decades with wide ranging social, environmental and economic impacts. Although the link between atmospheric circulation and meteorological drought is clear and often highlighted during major drought events, there is a lack of in-depth studies linking historical changes in meteorological drought indices and prevailing large-scale atmospheric patterns in Europe. To meet this shortfall, we investigated the relation between changes in large-scale atmospheric patterns and meteorological drought, as indicated by the geopotential height at 500mb (Z500) and the Standardised Precipitation-Evapotranspiration Index (SPEI), respectively. Calculations were done separately for four climate regions (North, West, Central-East and Mediterranean) over the growing season (March–September). Coherent patterns of significant changes towards higher pressure (increasing Z500) and drier conditions (decreasing SPEI) over 1979–2021 are found over West in spring and Central-East in summer. Z500 and SPEI are strongly linked, reflected by both significant (1979–2021) correlations and high co-occurrences (69-96%) between meteorological drought and high-pressure anomaly occurrences since 1900. North shows the most heterogeneous trend patterns and weakest links, but constitutes a hotspot of significantly increasing Z500 in September. Finally, we performed an ensemble-based, European wide analysis of future Z500, based on CMIP6 low-end (SSP126) and high-end (SSP585) 21st century emission scenarios. According to the projected changes, anomalously high-pressure systems will be the new normal regardless of scenario, and well exceeding the 2018 and 2022 levels in the case of the high-end emission scenario. However, due to the limitations of the model ensemble to represent the spatial heterogeneity in historical Z500 variability and trends (1979–2014), projected changes in large-scale circulation, and associated meteorological droughts, are highly uncertain. This paper provides new insight into significant trends in atmospheric circulation over Europe, their strong links to the observed drying trends, and the inability of a CMIP6 ensemble to reproduce the spatial heterogeneity of the circulation changes.

## Introduction

**Figure 1:**
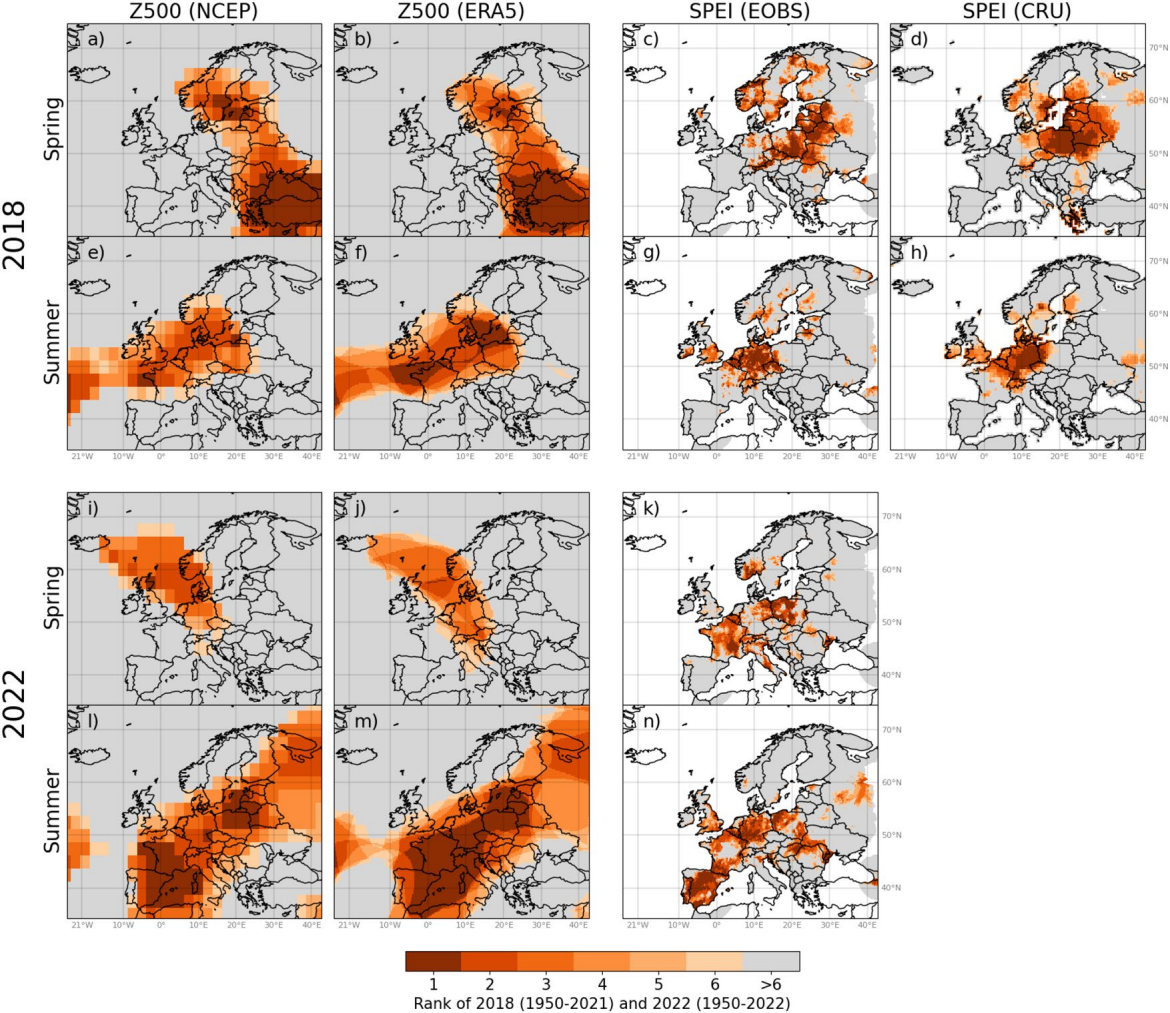
Top-six ranking of highest Z500 and lowest SPEI for spring 2018 (**a**–**d**), summer 2018 (**e**–**h**), spring 2022 (**i**–**k**) and summer 2022 (**l**–**n**). The period analysed is 1950–2021 for the 2018 event, and 1950–2022 for the 2022 event. A rank of one signifies that Z500 (SPEI) for a given season in 2018 or 2022 is record-breaking, i.e. the highest (lowest) during the analysed period. The figure was created in Python v3.7.3 (www.python.org), using the package Cartopy v0.17.0 (www.scitools.org.uk/cartopy/docs/v0.17/).

Over the last years the European continent has been struck by a series of extreme heatwaves coupled with long-lasting droughts, events that have put a strong pressure both on societal as well as on economic sectors. In 2018 and 2022, extreme to record-breaking meteorological drought and associated high-pressure-systems were observed over large areas in Europe (Fig. 1 and Supplementary Figs. S1–S4). These extreme events led to a long chain of severe impacts, including widespread wildfires, water supply issues, inland navigation restrictions, and substantially reduced crop yields and hydropower potential^1,2^.

Meteorological droughts, i.e. anomalously low meteorological water balance (precipitation minus potential evapotranspiration; P-PET), are typically driven by an anomaly in the large-scale atmospheric circulation, which is usually associated with blocking-like anticyclonic circulation centred over the drought-affected region^3–5^. Atmospheric blocking events are persistent, quasi-stationary high-pressure systems that interrupt the prevailing westerly flows and storm tracks^6^, being associated with a reduction in cloudiness, an increase in the mean air temperature and an overall rainfall deficit^5^. Higher temperatures lead to an increase in the potential evapotranspiration, which in turn causes higher actual evapotranspiration (if evaporative water is available) or an increase in the sensible heat flux due to less energy used for evapotranspiration^7^.

Geopotential height at 500mb (Z500) works as a steering level mirroring the prevailing tropospheric weather systems, and is commonly used when investigating the relationship between large-scale atmospheric circulation, heatwaves and meteorological drought^4,8–10^. Changes in Z500 are directly related to temperature changes between the surface and 500mb height (thermodynamic effect), whereas changes in the Z500 spatial patterns are affected by circulation changes (dynamical effect)^11,12^. Over the period 1979–2012, a decrease in the Z500 gradient between polar and mid-latitude regions in the Northern Hemisphere has been detected^13^, which in turn can moderate the westerly flow, with consequences especially for western European hydroclimate. A southward shift in the North Atlantic jet stream in summer has been detected, associated with more cyclonic storms (wet summers) across north-western Europe and fewer storms (and thus dry summers) over the Mediterranean^14,15^. Next to the large-scale atmospheric circulation, ocean circulation plays a prominent role in driving the hydroclimate variability^3,16^. A recent study related drying trends in central and southern parts of Europe to a slowdown of the Atlantic Meridional Overturning circulation (AMOC) through changes in the large-scale atmospheric circulation^3^. The combined effect of a slower AMOC and a higher frequency of atmospheric blocking circulation over the central part of Europe seems to be the perfect ingredient for driving long-lasting droughts over the European continent, in recent times as well as on longer time-scales^3,17,18^.

Meteorological droughts are already affected by climate change in every region of the world, including Europe^19^. Trends towards drier conditions in spring and summer have been detected in central, eastern and southern Europe, in line with increasing potential evapotranspiration since the 1970’s^20,21^. The frequency, duration and spatial extent of droughts, and extreme droughts in particular, are expected to increase in the 21st century under both moderate and high-end emission scenarios^22–24^, with the key causing factor being a further increase in the evapotranspiration^25^. However, a direct link to potential changes in the large-scale atmospheric circulation has not been explored in a systematic way. One reason may be that the newest generation of climate models (CMIP6) struggles to sufficiently produce blocking events^26,27^ and to properly detect any significant changes in the magnitude and location of the jet stream^28^. However, the representation of the prevailing large-scale atmospheric circulation has improved over the last generations of climate models^26,29^, and climate model ensembles are still the best sources of knowledge of what to expect of future changes in atmospheric circulation.

In-depth studies linking historical changes in prevailing large-scale atmospheric patterns and meteorological drought at the European level are lacking. To meet this shortfall, we used geopotential height at 500mb (Z500) and the Standardised Precipitation-Evapotranspiration Index (SPEI) to investigate Z500 as a driver of recent dryness trends and meteorological droughts events in Europe. Further, we analysed how the prevailing large-scale atmospheric circulation has changed over the past century, and how these changes will unfold in the future based on a low-end (SSP126) and a high-end (SSP585) emission scenario. We aimed to answer three main research questions (RQs): (1) What are the observed changes in spring and summer Z500 and SPEI over Europe during 1979–2021? (2) To what degree are regional variability in SPEI and Z500 (1979–2021), and their anomalies since the start of the 20th century, linked? (3) How is large-scale atmospheric circulation projected to change over the 21st century, and can we trust these projections? The focus was on Europe, which was separated into four main climate regions (North, West, Central-East and Mediterranean; Supplementary Fig. S5), over the growing season (March–September), i.e. the period of the year when drought impacts typically are most severe^30^. Multiple historical data sets were employed to increase the robustness of our results.

## Results

### Z500 and SPEI trends across Europe

**Figure 2:**
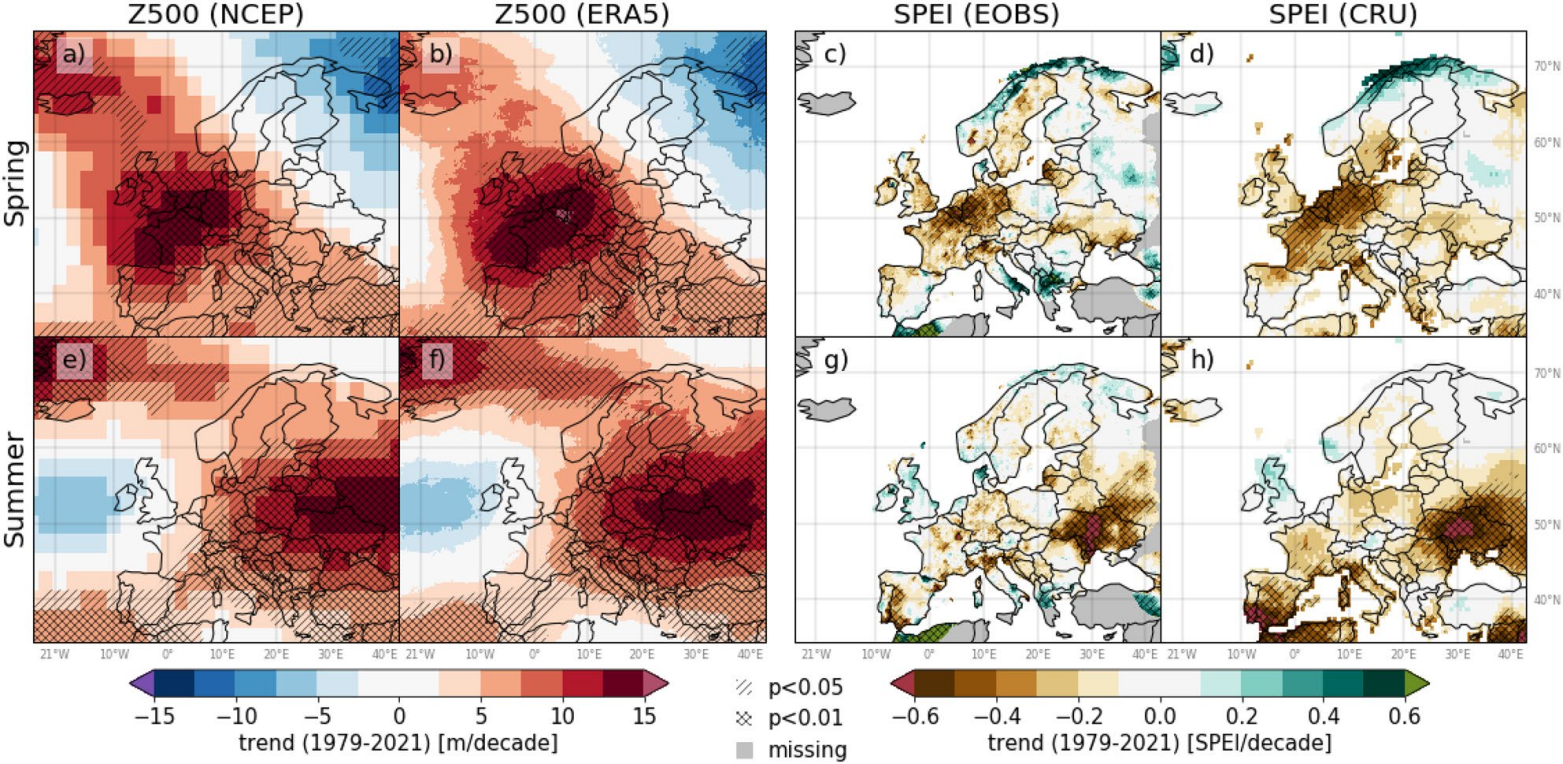
Trends in Z500 and SPEI for spring (**a**–**d**) and summer (**e**–**h**) over the period 1979–2021. The variable (data set) used in each column left to right: Z500 (NCEP), Z500 (ERA5), SPEI (EOBS) and SPEI (CRU). Single-hatching and double-hatching represent areas with statistically significant trends at 5% and 1% level, respectively. The figure was created in Python v3.7.3 (www.python.org), using the package Cartopy v0.17.0 (www.scitools.org.uk/cartopy/docs/v0.17/).

RQ1 was addressed by computing non-linear Z500 and SPEI trends and corresponding significances over 1979–2021 (Fig. 2 and Supplementary Fig. S6). Europe is dominated by pronounced and significantly increasing Z500 trends (towards higher pressure) in spring (March–May) and summer (June–August; Fig. 2). In spring, significantly increasing Z500 trends are seen across most of Europe, except in northeastern areas. The strongest spring trends in both increasing Z500 and decreasing SPEI (towards drier conditions) are located over western Europe (approx. − 10–10$^{\circ }$E, 40-60$^{\circ }$N). On a monthly basis (Supplementary Fig. S6), the strongest increasing Z500 trends exceed 15 m/decade. The strongest increasing Z500 trends over continental Europe during spring are found in April, whereas the strongest trends in March and May are located in the North-Atlantic Ocean. In March, a significantly decreasing trend in Z500 centred over northeastern Europe and parts of Russia is associated with increasing trends (wetter conditions) in SPEI in this area, as reflected in the seasonal trend.

In summer, a hotspot of strong and significantly increasing Z500 trends is found in eastern Europe (>10$^{\circ }$E). Correspondingly, a hotspot of strong significantly decreasing SPEI is found in eastern Europe Additionally, areas of significantly decreasing SPEI are seen in southwestern Europe, corresponding to significantly increasing Z500. July and August trends in Z500 and SPEI reflect the summer trends, with the strongest and most widespread significant drying trends found in August. The June trends imply higher pressure and drier conditions over large parts of eastern, central and western Europe.

Northeastern Europe is to a lesser degree affected by significantly increasing trends in Z500 during March–August, and have generally locally diverging or weak and non-significant SPEI trends throughout this period. However, it is at the centre of a hotspot of strong and significantly increasing Z500 trends in September. Despite these strong Z500 trends, no significant decreases in September SPEI over northeastern Europe are detected.

Overall, regions with the strongest (increasing) Z500 trends closely resemble the regions with the strongest (decreasing) SPEI trends, the exception being northeastern Europe in September. The two Z500 data sets (NCEP and ERA5) show a high correspondence in trend magnitude and significance. Whereas the spatial patterns in trends based on the two SPEI data sets (i.e., EOBS and CRU) are overall similar, the CRU data set typically depicts stronger and more widespread trends.

### Regional Z500 and SPEI trends

**Figure 3:**
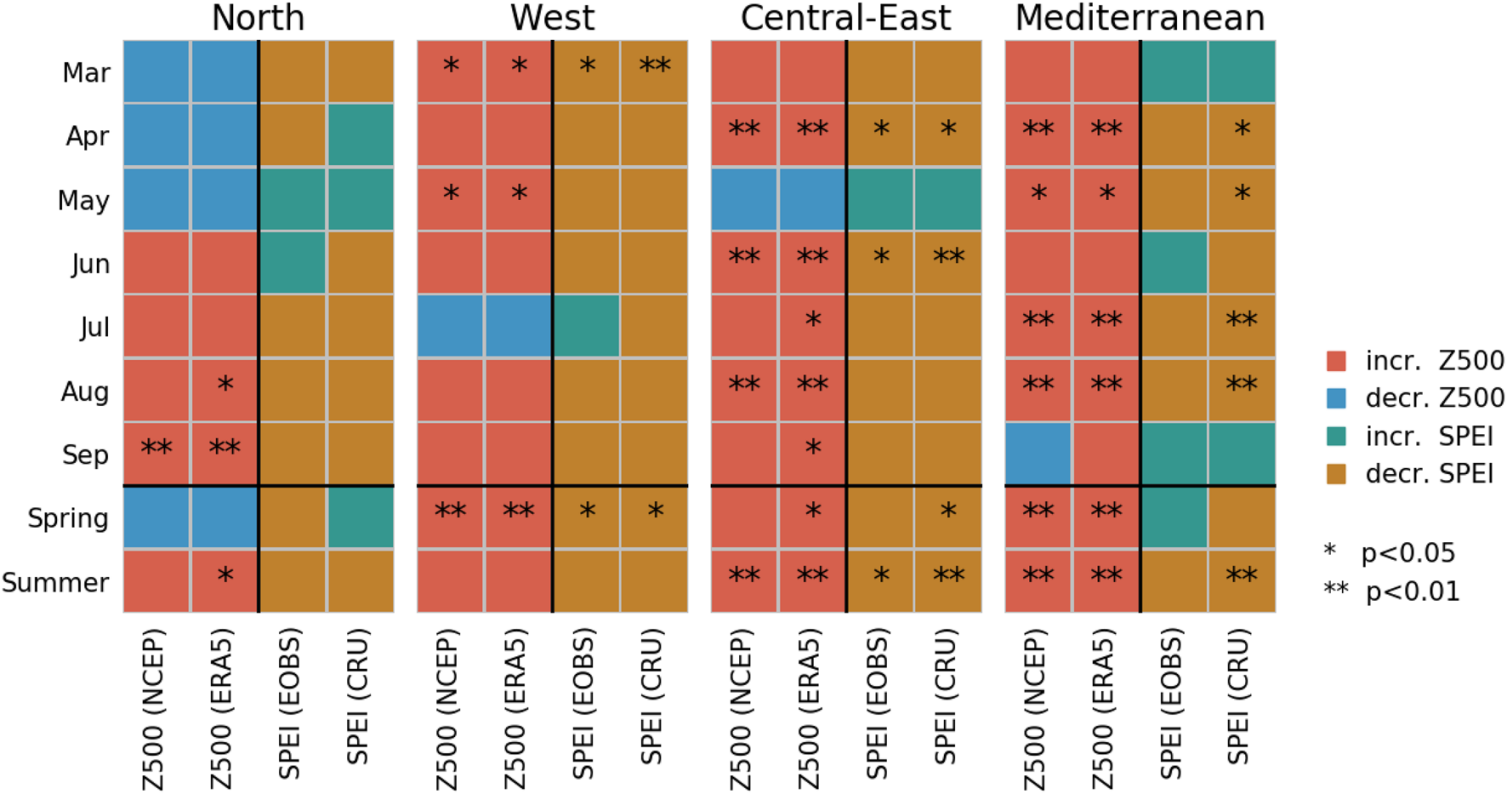
Trend directions of regional Z500 and SPEI over the period 1979–2021 for the months March–September, as well as spring and summer. One star and two stars represent significant trends at 5% level and 1% level, respectively.

Figure 3 summarises the monthly (March–September) and seasonal (spring and summer) trends in Z500 and SPEI for each of the European regions as defined in Supplementary Fig. S5: North (NO), West (WE), Central-East (CE) and Mediterranean (ME). The regional trends reflect grid-wise trends (Fig. 2 and Supplementary Fig. S6), but are averaged out due to spatial aggregation. Nevertheless, several significant regional trends (and coherent patterns) of increasing Z500 and decreasing SPEI are found. All regions are dominated by trends towards higher pressure and drier conditions. Notable, WE and CE show increasing Z500 and decreasing SPEI for the whole growing season except one month (July and May, respectively). NO shows the largest number of decreasing Z500 and increasing SPEI trends, especially in spring. Overall, a clear correspondence is found between the trend direction of Z500 trends and SPEI trends. All months and seasons with at least one significant trend in either Z500 or SPEI have corresponding increasing Z500 trends and decreasing SPEI trends for all data sets, except spring in ME. No significantly decreasing Z500 or increasing SPEI trends are found.

### Correlation between regional SPEI and Z500

Rank correlations between the regionally averaged Z500 and SPEI over 1979–2021 were computed to quantify the degree of relation between variabilities in SPEI and Z500 (RQ2). The results show significant correlations (at 1% significance level) for all regions and analysed seasons and months (Supplementary Fig. S7), confirming a strong correspondence between Z500 and SPEI. The highest correlations are found in WE, with all, but one, correlation coefficients being in the range [− 0.8, − 1]. NO has the overall lowest correlation coefficients. Z500 and SPEI correlations are higher in summer than spring in NO, and vice versa in CE, whereas they are more similar in WE and ME. Generally, the correlation coefficients are similar for a given time resolution (season or month) and region regardless of the data sets applied. Notable differences between the data sets are mainly found for SPEI. In these cases, SPEI (EOBS) has a higher correlation with geopotential height as compared to SPEI (CRU) in NO (in May and July) and WE (in September), vice versa in ME (in March, April, May, spring and summer), and mixed in CE (in March, May and August).

### Co-occurrence of regional high-pressure anomaly and meteorological drought

**Figure 4:**
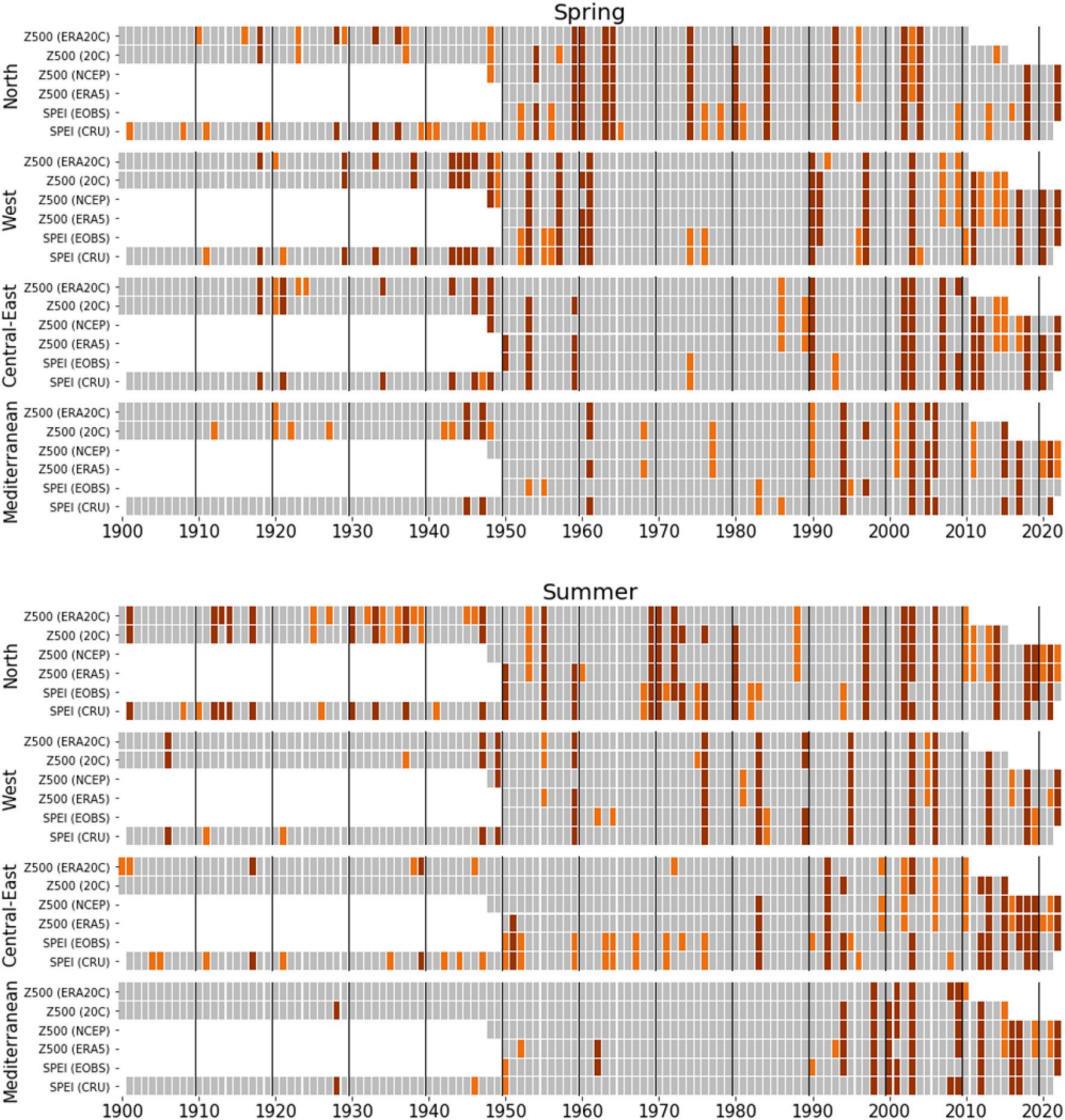
Regional 1900–2022 time series of anomaly occurrences in Z500 and SPEI for spring (top) and summer (bottom). Anomalies are coloured dark red if they co-occur for at least one Z500 and one SPEI data set, and orange otherwise. Anomaly occurrences are defined as years of Z500>80th percentile (high-pressure anomaly), and years of SPEI<20th percentile (meteorological drought). Percentiles are computed from the reference period 1981–2010. Grey boxes indicate years with data and no anomalies.

The co-variability of regional Z500 and SPEI (RQ2) was further addressed by looking at anomaly occurrences. Anomalies are here defined as high-pressure anomaly (>80th percentile Z500) and meteorological drought (<20th percentile SPEI), over the full period of each data record. Time series of the anomaly occurrences for spring and summer are shown in Fig. 4 (monthly March–September in Supplementary Figs. S8–S10), whereas the degree of co-occurrences between the time series are summarised in Supplementary Fig. S11.

The time series highlight seasons and months when high-pressure anomaly and meteorological drought co-occurred, such as the 1969–1970 summers (NO), the 2003 spring and summer (WE, CE and ME), the 2018 spring (NO and CE) and summer (NO, WE and CE), and the 2022 spring (NO, WE and CE) and summer (WE, CE and ME). High-pressure anomalies and meteorological droughts in spring and summer occur regularly over 1900–2022. However, a higher frequency of anomaly occurrences is apparent from the 1990’s onwards, in particular for ME and CE. In ME, only five (6%) of the summers before 1990 have co-occurring anomalies, compared to 17 (52%) of the summers over the period 1990–2022. During 2010–2022, summer high-pressure anomalies in CE are found for at least one of the data sets in all, but two years, with co-occurring meteorological drought in seven out of the 13 years. Co-occurrences between high-pressure anomaly and meteorological drought time series range from 69% to 96% (Supplementary Fig. S11). In most cases, the co-occurrences exceed 80%, even in the case of NO and CE, which overall have the lowest co-occurrences. In these regions, high-pressure anomalies and meteorological drought co-occur more often in spring than summer. In WE and ME, on the other hand, anomalies co-occur more often in summer than spring.

### Future Z500 projections

**Figure 5:**
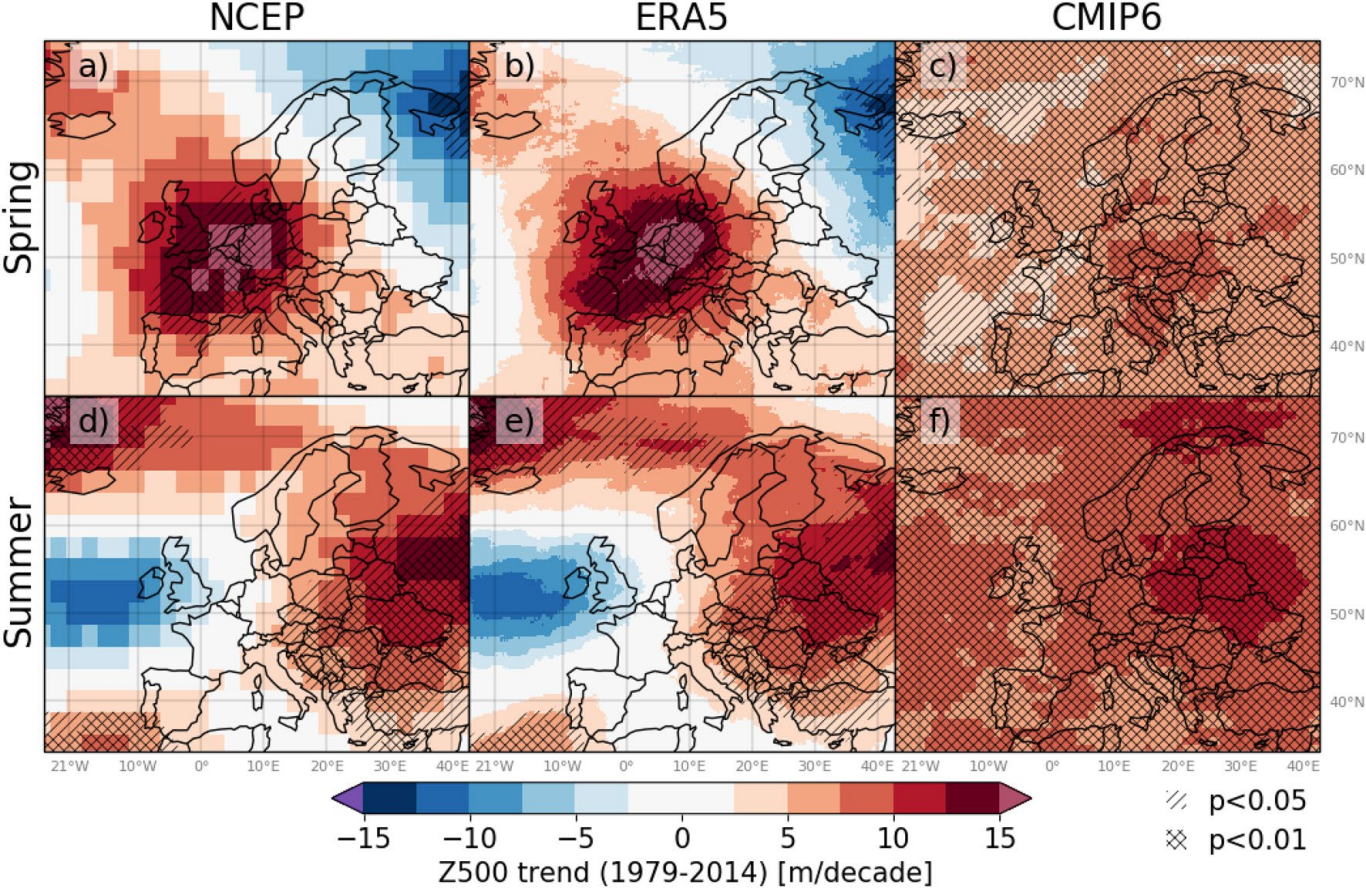
Gridwise Z500 trends in spring (**a**–**c**) and summer (**d**–**f**) over the period 1979–2014, using the data sets NCEP (left), ERA5 (middle) and CMIP6-Historical (right). Single-hatching and double-hatching represent areas with statistically significant trends at 5% and 1% level, respectively. The figure was created in Python v3.7.3 (www.python.org), using the package Cartopy v0.17.0 (www.scitools.org.uk/cartopy/docs/v0.17/).

RQ3 is addressed by i) evaluating Z500 trends computed by the historical CMIP6 ensemble mean (CMIP6-Historical) against the reanalysis data sets (NCEP and ERA5), and ii) computing regional Z500 anomaly time series of the low-end (SSP126) and high-end (SSP585) emission scenarios. The spatio-temporal variability of Z500 trends is notably lower in the CMIP6-Historical compared to the reanalysis data (seasonal and monthly trends in Fig. 5 and Supplementary Fig. S12, respectively). Unlike the rather spatially heterogeneous Z500 trends in NCEP and ERA5, significantly increasing Z500 trends are found across Europe and surrounding seas in all seasons and months by the CMIP6-Historical. The CMIP6-Historical Z500 trend values never exceeded 15 m/decade as found in the majority of the hotspots of increasing trends in the reanalysis data. Further, the regional Z500 anomaly time series (Supplementary Figs. S13–S18) reveal a low temporal variability in CMIP6-Historical as compared to the reanalysis data.

The increase in spring and summer regional CMIP5-Historical Z500 continues into the future according to both the SSP126 and SSP585 scenarios. However, the increase is more pronounced in the case of SSP585. The Z500 stabilises at magnitudes similar to the high-end anomalies of the reanalysis data in the second half of the 21st century for SSP126. According to SSP585, Z500 continues to increase until the end of the time series (i.e., 2100), with values well exceeding the most extreme historical high-pressure occurrences. This general pattern is found in all regions, seasons and months. The most extreme changes are found in ME, in which summer Z500 exceeds the most extreme historical high-pressure occurrences by the second half of the 21st century, even with the low-end emission scenario.

## Discussion

The extreme drought events of 2018 and 2022, with their wide range of environmental and socio-economic impacts, stress the importance of reducing costs and negative consequences of droughts. In order to do so, we need a deeper understanding of drought and its driving mechanisms at a regional and monthly to seasonal resolution. Due to the dynamical effect of atmospheric circulation, it is not sufficient to look at the direct effect of global warming. Whereas an increase in temperature directly affects the evapotranspiration component of meteorological drought, changes in the location of high-pressure systems are vital for understanding the spatial patterns of changes in meteorological drought. Nevertheless, near-surface temperature (in addition to precipitation) is by far the most widespread variable used when linking meteorological drought to climate change^22,31–34^. In this study, we aimed to meet this shortfall by analysing recent changes in the prevailing large-scale atmospheric circulation, as represented by changes in Z500, and link these changes to drying trends and meteorological drought events in Europe.

The 1979–2021 trend results imply higher pressure and drying over large parts of Europe. The Z500 trends are stronger over land than ocean, reflecting the role of positive soil moisture-atmosphere feedback^35–37^. Although Europe is dominated by increasing Z500 and decreasing SPEI trends (towards drier conditions), the spatial patterns of the significance, and even the direction of change, are heterogeneous. The dominance of corresponding increasing Z500 and decreasing SPEI trends reflects an overall warming trend, whereas the spatial heterogeneity in the trend magnitude and direction points to the role of atmospheric circulation dynamics. For Z500 in particular, a southwest (increasing) northeast (decreasing) divide in spring trends, and a west (decreasing) east (increasing) divide in summer trends are detected. These clear spatial patterns in seasonal Z500 trends resemble those previously found for 1979–2012^13^ (summer) and 1979–2013^38^ (spring and summer). In addition to an updated study period, the present study adds to the previous Z500 trend results by using a more robust trend method (not assuming any parametric distribution of the data), and by adding information about the trend significance. The divide between west and east in the trend signals supports the division into four regions based on the Köppen-Geiger climate classification.

The Z500 and SPEI trends over Europe are closely linked. Overall, the spatio-temporal trend patterns of SPEI follow those of Z500 when the Z500 trends are significant. The hot spots of significantly decreasing SPEI over western Europe in spring and eastern Europe in summer have a stronger west-east divide as compared to trends spanning further back in time. For example, decreasing summer P-PET in large areas from southwest to eastern Europe is detected over the period 1950–2018^13^. Similarly, significantly decreasing SPEI is found in large areas from southwest to eastern Europe in both spring and summer over the period 1902–2019^20^. Notably, the same study^20^ found no spring trends in the areas depicting the strongest decreasing spring trends in the present study, indicating that the drying trend in this area is a rather recent phenomenon. The averaging of decadal variability in atmospheric circulation dynamics may be the reason for the relatively more uniform spatial patterns found in studies using longer time periods.

The only area of significantly decreasing Z500 trends is found in the North region (NO) in spring, in particular in March. The SPEI trends are locally heterogeneous in NO, and are generally non-significant (except for spring). Decreasing Z500 and increasing SPEI in NO point to the role of circulation changes. From a thermodynamic viewpoint, one would expect stronger Z500 increases and SPEI decreases in NO as compared to further south due the relatively stronger warming in the north (i.e. Arctic amplification)^39^. Spring trends towards decreasing atmospheric blocking frequency and intensity have been detected over parts of northeastern Europe over 1979–2019^40^, and may in part explain the observed decreasing Z500 trends. However, depending on the applied blocking methodology, decreasing trends in blocking frequency and intensity are also detected in areas of increasing Z500 trends in spring and summer^40^. NO stands out in September when it is dominated by a widespread hotspot of increasing Z500 trends. The low correspondance between Z500 and SPEI September trends in NO may be explained by the local variability in hydrometeorology and topography in this region.

The coherent spatiotemporal patterns in Z500 and SPEI trends are confirmed by the significant correlations found for all regions, seasons and months. Despite an overall strong link between Z500 and SPEI, notable regional differences are found. The strongest correlations were found in WE. This is in line with circulation changes having a stronger effect on changes in temperature and precipitation in northwestern Europe than in southeastern Europe^12^. Pressure systems over western Europe are typically stable for a longer time in April^9^, which may explain the particularly strong correlations in WE for this month. April is also the spring month with the largest areas of significantly increasing Z500 and decreasing SPEI over continental Europe, in line with its particular susceptibility to drying driven by an increase in the frequency of atmospheric blocking over the North Sea and central part of Europe^9^.

The link between the high-pressure and meteorological drought are further demonstrated by the high co-occurrence in the anomaly occurrence time series, and also demonstrated for the record-breaking events of 2018 and 2022. Whereas the anomaly occurrence time series focused on co-occurring (moderate to extreme) high-pressure and meteorological drought over multiple decades, the 2018 and 2022 events depict their co-occurrence over the growing season in extreme years. Although clear increases in anomaly occurrences from the 1990’s are apparent in the Mediterranean (ME) and Central-East (CE), multiple anomaly occurrences in earlier decades are found in NO and (to a lesser degree) WE. Thus, both circulation dynamics and recent regional warming affect the temporal patterns of high-pressure and meteorological drought occurrences.

A high agreement is found between the different data sets of Z500 for all regions and time scales, both in the location and magnitude of the trends and in the high-pressure anomaly occurrences. The minor differences found using NCEP versus ERA5 mainly result from the different spatial resolutions of the two data sets. At the regional scale, a high agreement is also found for the two SPEI data sets. However, differences in SPEI trends are observed at the sub-regional level, in particular in areas outside the trend hotspots. A likely reason for this difference is the different PET estimation methods in the two data sets, i.e. the Penman-Monteith method in CRU and the Hargreaves method in EOBS. The Penman-Montheith PET has shown to have a stronger trend over Europe as compared to the Hargreaves PET^21^, which particularly proved to be true for the ME region (Supplementary Fig. S19).

Future climate projections, based on the most recent climate models (i.e., CMIP6), indicate that Z500 will increase in all European regions. Whether or not this implies an increase in drought severity is unclear as a heightened Z500 may not necessarily have anticyclonic characteristics in a warmer Europe. According to SSP126, Z500 will stabilise by the second half of the 21st century at magnitudes similar to the historical high-pressure anomalies. Under the most pessimistic scenario (SSP585), Z500 are not expected to stabilise within the 21th century, in line with previous studies^38,41,42^. Rather, it will reach levels not apparent in any of the reanalysis data records (longest extending back to 1836), well exceeding the high-pressure anomalies of 2018 and 2022. However, these projections are highly uncertain, as the ability of CMIP6-Historical to properly simulate interannual variability and changes in Z500 is overall weak.

Our study detect coherent spatiotemporal trend patterns and significant correlations between Z500 and SPEI over Europe. Further, a strong relation between high-pressure systems and meteorological drought was confirmed by a high degree of co-occurring regional anomalies since the beginning of the 20th century, and during the extreme events of 2018 and 2022 in particular. Our findings imply that meteorological droughts are sensitive, not only to the thermodynamic effect of global warming, but also to changes in the prevailing large-scale atmospheric circulation. This limits the trust in future drought projections as the current generation of climate models have a limited ability to represent circulation dynamics.

## Methods

### Geopotential height at 500mb (Z500) and meteorological drought index (SPEI)

The applied data sets are summarised in Table 1. The data sets comprise four reanalysis data sets of geopotential height at 500mb (Z500), three model ensemble mean data sets of Z500 (one historical period, and two future scenarios), and two gridded observational data sets used for calculating the meteorological drought index SPEI. We employed multiple historical data sets to assess the uncertainty of the results arising from the underlying data set.

**Table 1:** Data sets of Geopotential Height at 500mb (Z500) and Standardised Precipitation-Evapotranspiration Index (SPEI).

Variable	Data set abbreviation	Data set	Period	Spatial resolution
Z500	NCEP	NCEP/NCAR reanalysis 1^43^	1948–2022	2.5$^\circ$
Z500	ERA5	ERA5 reanalysis^44,45^	1950–2022	0.25$^\circ$
Z500	ERA20C	ERA-20C reanalysis^46^	1900–2010	1.4$^\circ$ $^*$
Z500	20C	NOAA-CIRES-DOE 20th century reanalysis version 3^47^	1836–2015	1$^\circ$
Z500	CMIP6-Historical	CMIP6 model ensemble mean for historical period (Table 2)	1850–2014	1$^\circ$
Z500	CMIP6-SSP126	CMIP6 model ensemble mean for SSP126 (Table 2)	2015-2100	1$^\circ$
Z500	CMIP6-SSP585	CMIP6 model ensemble mean for SSP585 (Table 2)	2015-2100	1$^\circ$
SPEI	EOBS	E-OBS gridded daily observations version 25.0e^48^	1950–2022	0.1$^\circ$
SPEI	CRU	CRU TS gridded monthly observations version 4.06^49^	1901–2021	0.5$^\circ$

1.4$^\circ$ $^*$: X axis: whole world in 256 1.41$^\circ$ steps, first point at 0.00$^\circ$ E, last point at 358.59$^\circ$ E. Y axis: Gaussian grid with 128 steps, first point at 88.93$^\circ$ N, last point at 88.93$^\circ$ S.

#### Z500 from reanalysis

The four reanalysis data sets included the NCEP-NCAR (National Centers for Atmospheric Prediction and the National Center for Atmospheric Research) Reanalysis 1 (NCEP)^43^, the fifth generation ECMWF (European Centre for Medium-Range Weather Forecasts) atmospheric reanalysis (ERA5)^44,45^, the ECMWF’s atmospheric reanalysis of the 20th century (ERA20C)^46^, and NOAA-CIRES-DOE (National Oceanic and Atmospheric Administration - Cooperative Institute for Research In Environmental Sciences - Department of Energy) 20th Century Reanalysis version 3 (20C)^47^. The reanalysis data sets provide global spatially and temporally uniform representation of the atmospheric state. Despite known errors from the underlying models and assimilated observational data, the most recent reanalysis data sets have undergone significant improvements that make them useful for examination of large-scale changes in Z500^13^.

Period covered and spatial resolutions are given in Table 1. All data sets have monthly mean values. ERA5 and ERA20C provide the geopotential (m$^2$s$^{-2}$), and Z500 (m) was computed by dividing the geopotential by the Earth’s gravitational acceleration (9.80665ms$^{-2}$). The domain − 30–50$^\circ$ longitude and 30–80$^\circ$ latitude (Europe) was selected.

#### Z500 from climate models

We applied three data sets of modelled ensemble mean Z500 from the Coupled Model Intercomparison Project phase 6 (CMIP6)^65^: the historical period (1850–2014; CMIP6-Historical) and the Shared Socioeconomic Pathways 1-2.6 (CMIP6-SSP126) and 5-8.5 (CMIP6-SSP585). SSP126 and SSP585 represent the low-end and high-end emission scenarios, respectively^66,67^. The emission scenarios are anticipated to produce a radiative forcing in 2100 of approximately 2.6 Wm$^{-2}$ in SSP126 due to an increasing shift toward sustainable practices, and 8.5Wm$^{-2}$ in SSP585 due to a fossil-fuel driven development. Table 2 provides details and references of the 15 models comprising the model ensembles. The CMIP6 model outputs were downloaded from the Earth System Grid Federation (ESGF) at https://esgf-node.llnl.gov/search/cmip6/ (last accessed 31.10.2020). The model data were regridded to a shared 1$^\circ$ resolution. We considered all ensemble members from each model available at the time of the study.

**Table 2:** Models and number of applied model realizations for the Historical, SSP126 and SSP585 CMIP6 scenario ensembles.

Model	Historical	SSP126	SSP585
ACCESS-CM2^50^	2	1	1
ACCESS-ESM1-5^51^	3	3	3
AWI-CM-1-1-MR^52^	2	1	1
CanESM5^53^	25	25	24
EC-Earth3^54^	3	1	1
EC-Earth3-Veg^55^	2	3	3
GFDL-ESM4^56^	3	1	1
INM-CM4-8^57^	1	1	1
INM-CM5-0^58^	10	1	1
IPSL-CM6A-LR^59^	32	6	6
MIROC6^60^	10	3	3
MPI-ESM1-2-HR^61^	7	2	2
MPI-ESM1-2-LR^62^	10	10	10
MRI-ESM2-0^63^	5	1	1
NESM3^64^	5	2	2

#### SPEI from observations

A widely used indicator of meteorological drought is the Standardised Precipitation-Evapotranspiration Index (SPEI) over a given accumulation period (e.g. SPEI3 for a 3-month accumulation period^68^). SPEI measures the normalised anomalies in the climatic water balance defined as precipitation (P) minus potential evapotranspiration (PET). The most precise PET estimate is calculated using the Penman-Montieth (P-M) PET equation^69^, which requires high-quality radiation, humidity, wind and temperature data. In cases where the required data are not available, the Hargreaves equation^70^ has proven a useful balance between consistency and minimal data requirements^71^. The Hargreaves equation uses a temperature-proxy method for daily net radiation and estimates (extraterrestrial) radiation based on the latitude and day of the year. Consequently, the meteorological data required for estimating PET is reduced to daily data of mean, minimum and maximum temperature.

We calculated SPEI both using the E-OBS^48^ and Climate Research Unit (CRU) v4.06^49^ data sets. E-OBS is based on the European Climate Assessment and Dataset station information (ECA &D), and comprises Europe-wide daily meteorological variables from 1950 on a 0.1$^\circ$ longitude/latitude grid. We applied E-OBS v25.0e that have data until December 2021, and the monthly E-OBS updates of 2022. The CRU time series data comprise monthly 1901–2021 climate data at a 0.5$^\circ$ spatial resolution. From CRU, precipitation and the readily available P-M based PET estimates^69,72^ with the same spatial coverage as P, were used to compute precipitation minus potential evapotranspiration (P-PET). In terms of E-OBS, the spatial and temporal coverage of the variables needed to calculate P-M PET (and thus the readily available E-OBS v23.1 P-M based PET index from 1981; Supplementary Fig. S19) was lower than that of the temperature data. Consequently, the Hargreaves equation was used to estimate PET from the E-OBS data set using daily mean, minimum and maximum air temperature. We performed a regional comparison of the E-OBS based PET estimates using P-M and Hargreaves to evaluate our choice (Supplementary Fig. S19). The Hargreaves method generally provides higher PET estimates than the P-M method. They agree on the trend significance in all seasons and most months. In the Mediterranean region, the P-M consistently shows stronger trends than HA, in line with previous comparison of the two methods over Europe^21^. Even though more trust should be given the P-M method, the extra data required by the P-M are often of less quality. Thus, it is not straightforward to conclude on the best PET method. However, the correlation between the two are very high (>90) for spring and summer. This gives us trust that the E-OBS based Hargreaves PET is a sufficient estimate for our analysis. From daily precipitation (P) and PET, monthly mean P-PET was computed.

The E-OBS based SPEI was calculated by the following procedure using the R-package *SCI*^73^. For each month (March–September) and season (spring and summer) separately, P-PET over the reference period 1981–2010 was fitted to the generalised extreme value (GEV) distribution following the recommendations by^74^. Non-exceedance probabilities from the GEV distribution were transformed to the standard normal distribution, which were used to estimate the SPEI in terms of standard deviations for each grid cell and time step. The reference period (1981–2010) was chosen as the most recent 30-year period covered by all data sets of SPEI and Z500, in order to have a consistent reference period throughout the analysis. Because the main purpose of our analysis is to evaluate changes and relative extremes in SPEI, and not the exact SPEI values as such, similar results are expected regardless of the applied reference period.

### Analyses

To answer RQ1, Z500 and SPEI trends, corresponding significances, and anomaly (high-pressure and meteorological drought occurrences) time series were computed. RQ2 is addressed by computing the rank correlation between the two variables and the percentage of co-occurrence between the time series of high-pressure anomaly and meteorological drought occurrences. Anomaly time series of future scenario Z500 were computed to answer RQ3. The months March to September were selected to represent the growing season when most impacts of droughts are normally found. It is also a period of typically snow-free conditions in regions subject to seasonal snow (with the exception of high latitude and altitude regions). Analyses were performed on the seasonal means of spring (March–May) and summer (June–August), as well as for each month separately. Z500 and SPEI were derived for the same temporal scale, i.e. the mean seasonal Z500 and SPEI with a three-month accumulation period (SPEI-3) for May, respectively August, and the mean monthly Z500 and SPEI with a one-month accumulation (SPEI-1). Historical trends and correlations were calculated for the period 1979–2021, whereas the full period of each data set was used for anomaly occurrences. As for the SPEI calculations, the reference period 1981–2010 was used to derive the anomaly percentiles values.

#### Z500 and SPEI trends across Europe

Monthly and seasonal trends in Z500 and SPEI were calculated using the non-parametric Sen’s slope (or Kendall-Theil robust line)^75^ and the corresponding significance obtained by the Mann-Kendall test^76,77^ using the *pyMannKendall* package in Python^78^. The trends were calculated over the satellite era period (1979–2021). The 1970’s also marks a starting point of increasing temperature and potential evapotranspiration in Europe^20,21^. Two Z500 data sets (NCEP and ERA5) and two SPEI data sets (EOBS and CRU) covering the same period were used. As the CRU data set only was available until the end of 2021, the trends were limited to this period.

#### Defining European regions

To produce area-weighted time series for the regional analysis, Europe was divided into four climate regions (Supplementary Fig. S5). The regions were based on the Köppen-Geiger climate classification^79^:

- North (NO): Dominated by the climate type Dfc, signifying a cold climate without a dry season and with cold summers.
- West (WE): Dominated by the climate type Cfb, signifying a temperate climate without a dry season and with warm summers.
- Central-East (CE): Dominated by the climate type Dfa, signifying a cold climate without a dry season and with hot summers.
- Mediterranean (ME): Dominated by the climate types Csa and Csb, signifying a temperate climate with dry and hot (Csa) or warm (Csb) summers.

Grid cells on the border between regions were allocated to the region that included the grid cell’s centre point. In cases where the centre points were exactly at a regional divide, the grid cells were allocated to the lowermost and/or leftmost region to ensure no grid-cells belonged to more than one region. The stippled lines defining the regions in Supplementary Fig. S5 illustrate the variability in the regional boundaries of the different data sets. Gridded SPEI values outside the range [-3, 3] were truncated prior to calculating the mean regional time series to avoid large biases to outliers^74^.

#### Regional Z500 and SPEI trends and correlations

Trends over 1979–2021 were computed using area-weighted regional time series. Z500 and SPEI regional trends were compared using the direction of the trends and two significance levels (1% and 5%). The 1979–2021 Spearman rank-order correlation coefficient and corresponding p-value were calculated for each combination of Z500 (NCEP or ERA5) and SPEI (E-OBS or CRU) time series using the *scipy.stats* method spearmanr in Python^80,81^. The Spearman correlation has no parametric assumption, and was therefore preferred over the traditional Pearson correlation, which assumes normally distributed data. Similar to the Pearson correlation coefficient, correlations of $+$1 and −1 signify an exact monotonic relationship, and zero that there is no correlation.

#### Co-occurrence of regional high-pressure anomaly and meteorological drought

Whereas rank correlation is a measure of the general co-variability in Z500 and SPEI, independent of the state of the variables, we also wanted to investigate the co-occurrence of high-pressure anomaly (high end of Z500) and meteorological drought (low end SPEI) specifically. The 20th percentile (P20) of SPEI and 80th percentile (P80) of Z500 based on the reference period, was derived for each data set. Values more extreme than the given percentile threshold were defined as anomaly occurrences, i.e. high-pressure anomaly (in the case of Z500>P80) and meteorological drought (in the case of SPEI<P20). Time series of anomaly occurrences were plotted using the full period available for each data set. The percentages overlap were computed to estimate the co-occurrence of the different anomaly occurrence time series.

#### Link between Z500 and SPEI under extreme drought events

To assess the link between meteorological drought and atmospheric circulation under extreme conditions, we calculated the rank of Z500 and SPEI in the extreme drought years 2018 and 2022. We selected the common period (1950–2021) for Z500 (NCEP), Z500 (ERA5), SPEI (EOBS) and SPEI (CRU) as the basis for the ranking of the 2018 event. A rank of one signifies that the average Z500 or SPEI for a given month or season in 2018 is the highest, respectively lowest, in the whole period 1950–2021. Because the CRU data for 2022 were not available at the time of analyses, the ranks of the 2022 event were calculated for Z500 (NCEP), Z500 (ERA5) and Z500 (EOBS) based on the period 1950–2022. Both grid-wise and regional ranks were computed.

#### Future Z500 projections

Regional time series of historical and future changes in geopotential height were derived using the reanalysis data sets and CMIP6 ensemble mean Z500 historical, SSP126 and SSP585 scenarios. Anomaly time series were calculated by subtracting the 1981–2010 mean. For the CMIP-SSP126 and CMIP6-SSP585 time series, the 1981–2010 mean of the corresponding CMIP-historical time series was used. To evaluate the modelled Z500 trends, we compared the grid-wise trends in the CMIP-Historical and the reanalysis. Because CMIP-Historical ends in 2014, trends were compared for the period 1979–2014.

### Supplementary Information

Supplementary Figures.

## Data Availability

Our study is based on freely available third-party data. All reanalysis data (NCEP, ERA5, 20C and ERA20C) are available at https://climexp.knmi.nl/selectfield_rea.cgi?id=someone@somewhere, E-OBS data at https://surfobs.climate.copernicus.eu/dataaccess/access_eobs.php, CRU data at https://crudata.uea.ac.uk/cru/data/hrg/cru_ts_4.06/cruts.2205201912.v4.06/, and CMIP6 model outputs at https://esgf-node.llnl.gov/search/cmip6/.
