# Supplementary material for: Recent European drying and its link to prevailing large-scale atmospheric patterns
Source: Sci Rep. 2023 Dec 8;13:21921. doi: 10.1038/s41598-023-48861-4 (PMC10713605; doi:10.1038/s41598-023-48861-4)
Supplement: Supplementary file 1 — Supplementary Figures. [file 41598_2023_48861_MOESM1_ESM.pdf]

Supplement to "Recent European drying and its link to prevailing large-scale atmospheric patterns"

Sigrid J. Bakke, Monica Ionita, and Lena M. Tallaksen

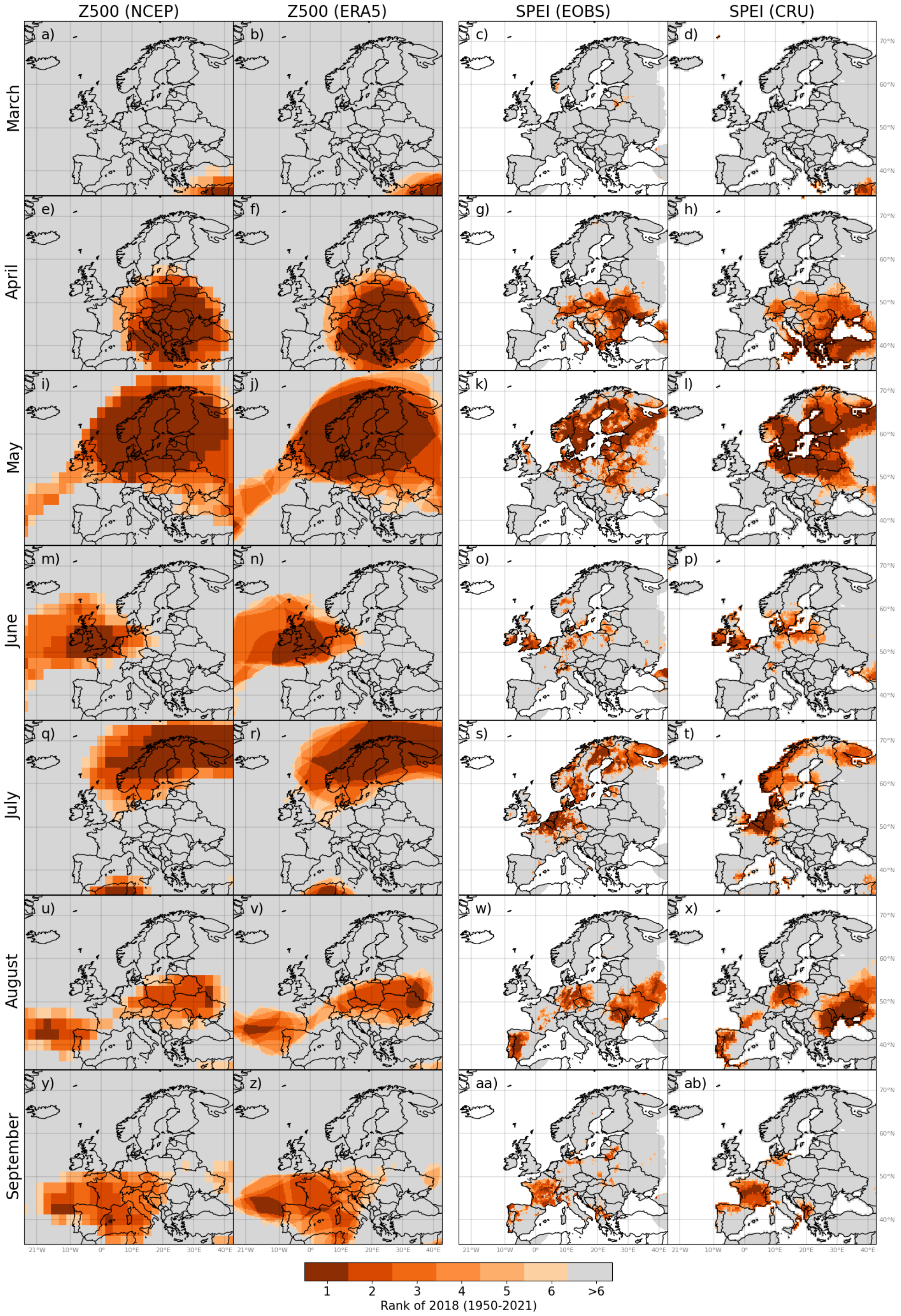

Figure S1: Top-six ranking of highest Z500 and lowest SPEI for the months March–September 2018. Analysed period is 1950–2021. A rank of one signifies that Z500 (SPEI) for a given month of 2018 is the highest (lowest) during the analysed period. The figure was created in Python v3.7.3 (`www.python.org`), using the package Cartopy v0.17.0 (`www.scitools.org.uk/cartopy/docs/v0.17/`).

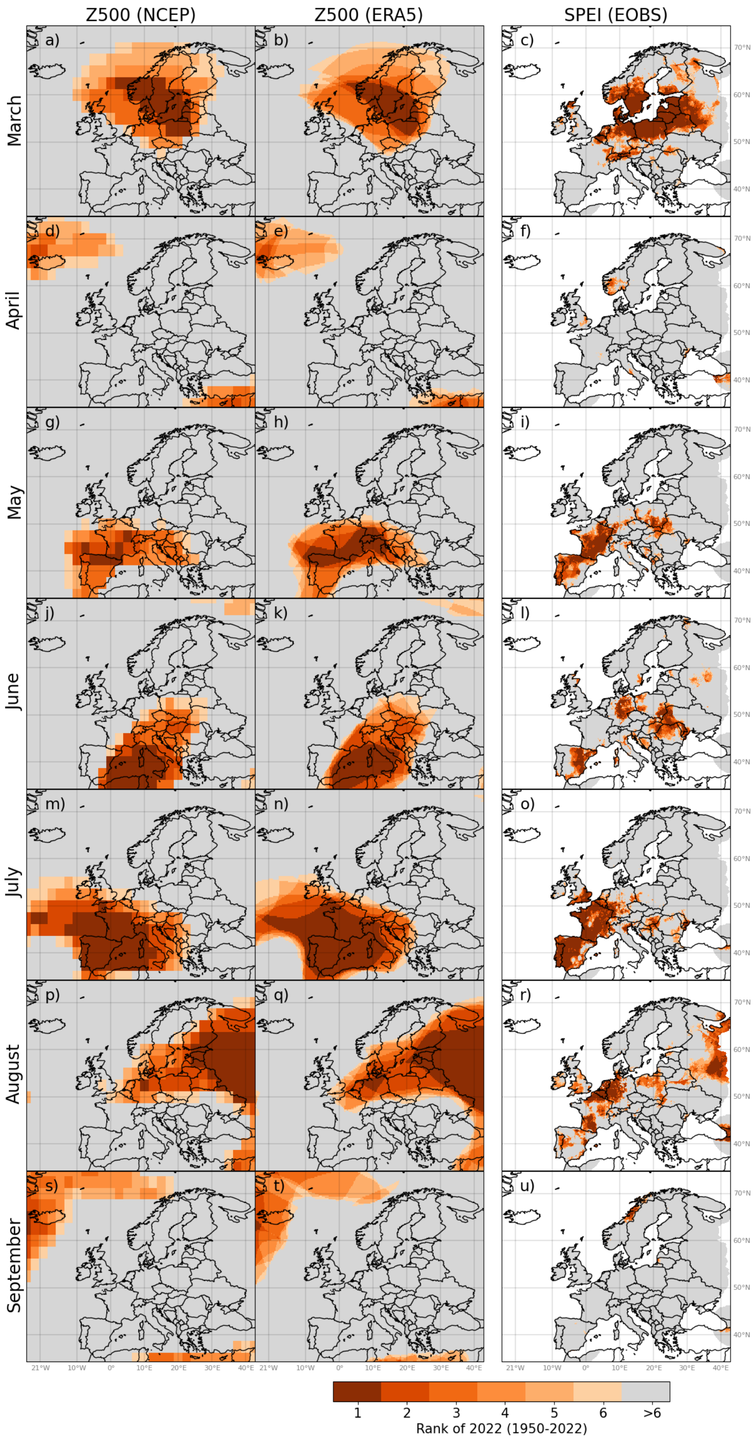

Figure S2: Top-six ranking of highest Z500 and lowest SPEI for the months March–September 2022. Analysed period is 1950–2022. A rank of one signifies that Z500 (SPEI) for a given month of 2022 is the highest (lowest) during the analysed period. The figure was created in Python v3.7.3 (`www.python.org`), using the package Cartopy v0.17.0 (`www.scitools.org.uk/cartopy/docs/v0.17/`).

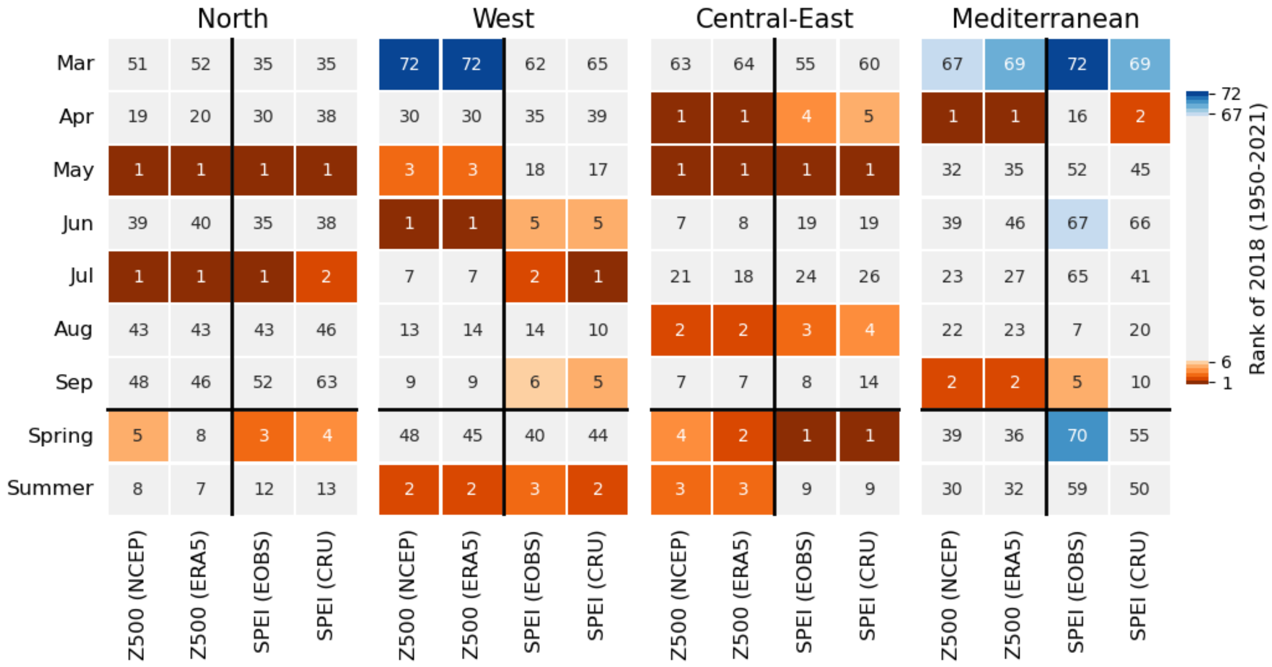

Figure S3: Ranking of regional Z500 and SPEI in each month March–September, as well as spring and summer of 2018. Analysed period is 1950–2021. The six most extreme ranks on both the high and low end are coloured. A rank of one signifies that the average Z500 (SPEI) for a given region and period of 2018 is the highest (lowest) during the analysed period. Vice versa, a rank of 72 signifies that the average Z500 (SPEI) for a given region and period of 2018 is the lowest (highest) during the analysed period. Regions are defined as given by Fig. S5.

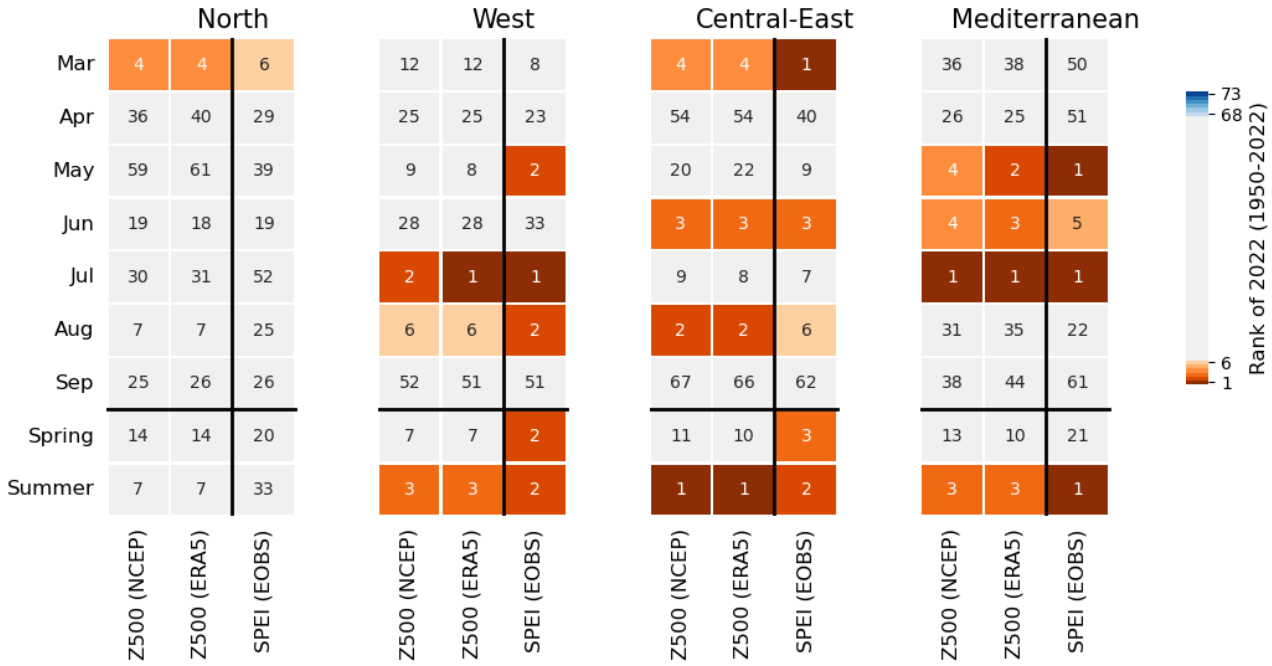

Figure S4: Ranking of regional Z500 and SPEI in each month March–September, as well as spring and summer of 2022. Analysed period is 1950–2022. The six most extreme ranks on both the high and low end are coloured. A rank of one signifies that the average Z500 (SPEI) for a given region and period of 2022 is the highest (lowest) during the analysed period. Vice versa, a rank of 73 signifies that the average Z500 (SPEI) for a given region and period of 2022 is the lowest (highest) during the analysed period. Regions are defined as given by Fig. S5.

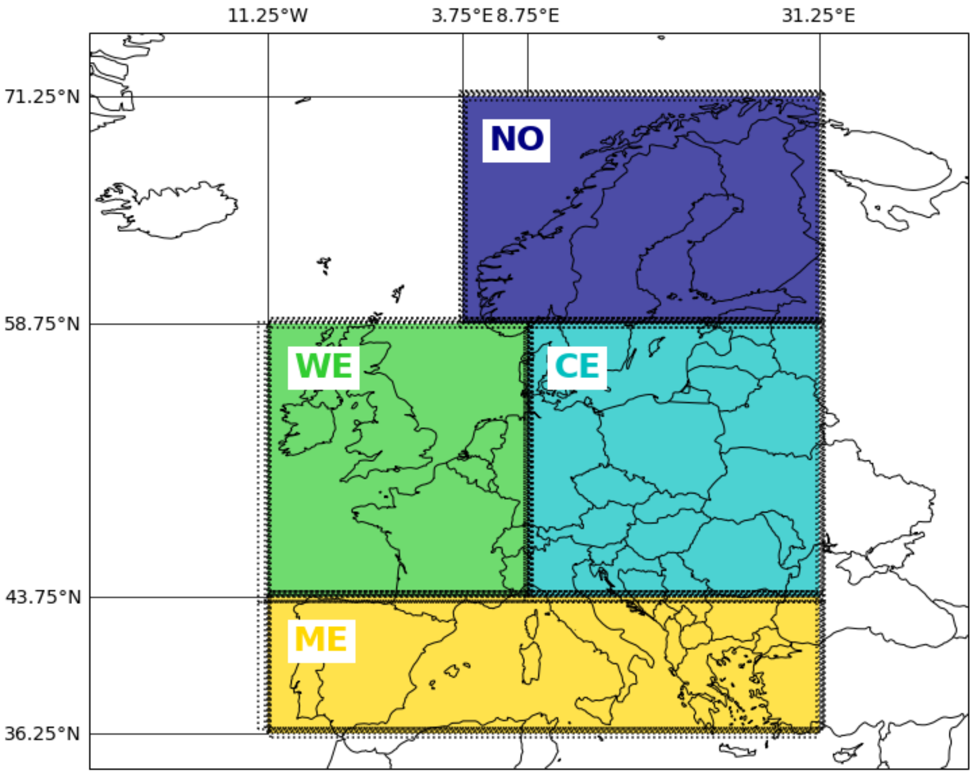

Figure S5: Regions (colours) defined based on the Köppen-Geiger climate classification. The regions are called North (NO; dark blue), West (WE; green), Central-East (CE; light blue) and Mediterranean (ME; yellow). The domain of each region is defined based on the longitudinal and latitudinal coordinates given along the x-axis and y-axis, respectively. The stippled black lines close to the region boundaries represent the region boundaries given by the different data sets, which vary slightly due to their different spatial grids. The figure was created in Python v3.7.3 (`www.python.org`), using the package Cartopy v0.17.0 (`www.scitools.org.uk/cartopy/docs/v0.17/`).

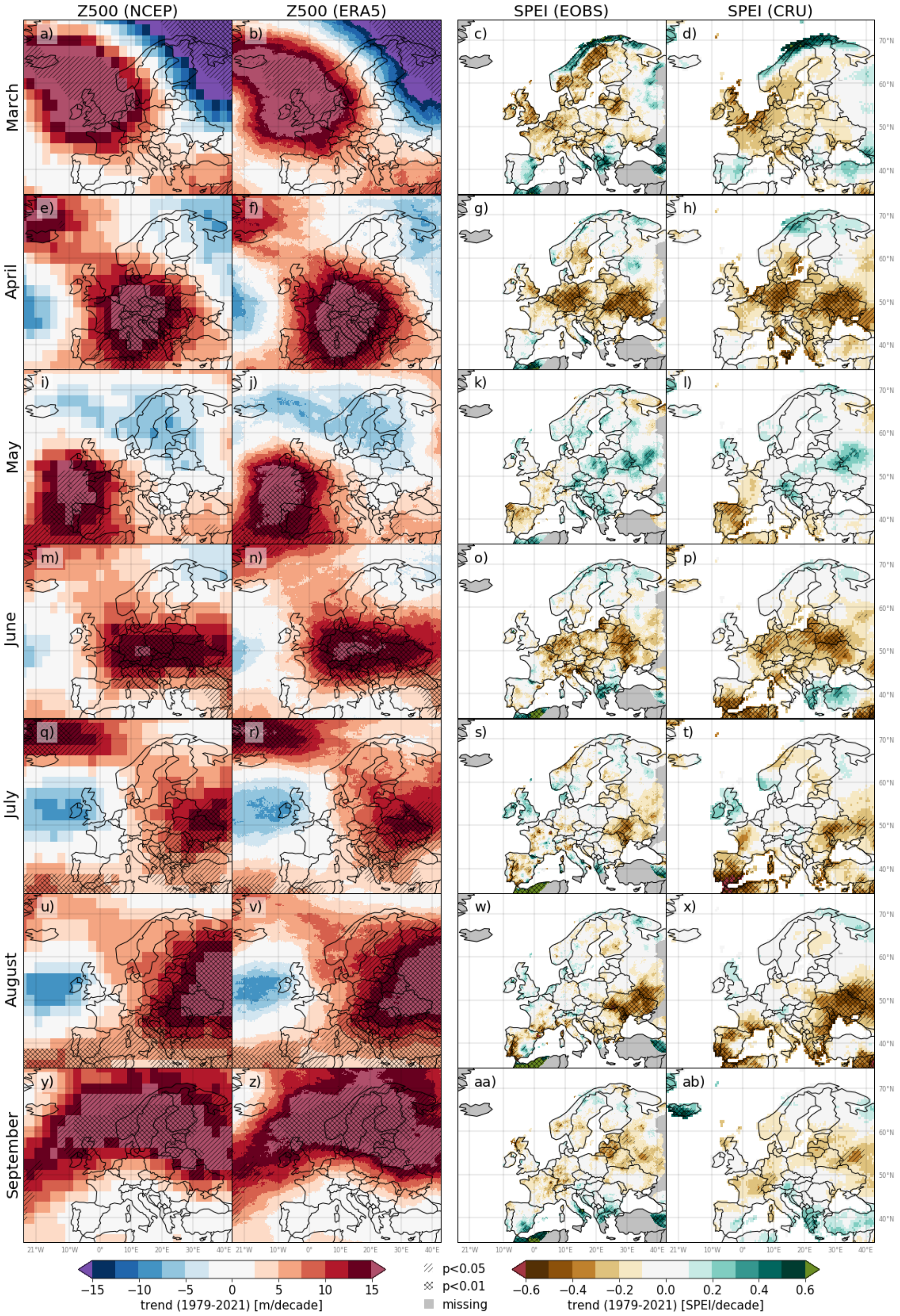

Figure S6: Gridwise trend for Z500 and SPEI over 1979–2021 for each month March (top) to September (Bottom). Variable (data set) used in each column left to right: Z500 (NCEP), Z500 (ERA5), SPEI (EOBS) and SPEI (CRU). Single-hatching and double-hatching represent areas with statistically significant trends at 5% and 1% level, respectively. The figure was created in Python v3.7.3 (`www.python.org`), using the package Cartopy v0.17.0 (`www.scitools.org.uk/cartopy/docs/v0.17/`).

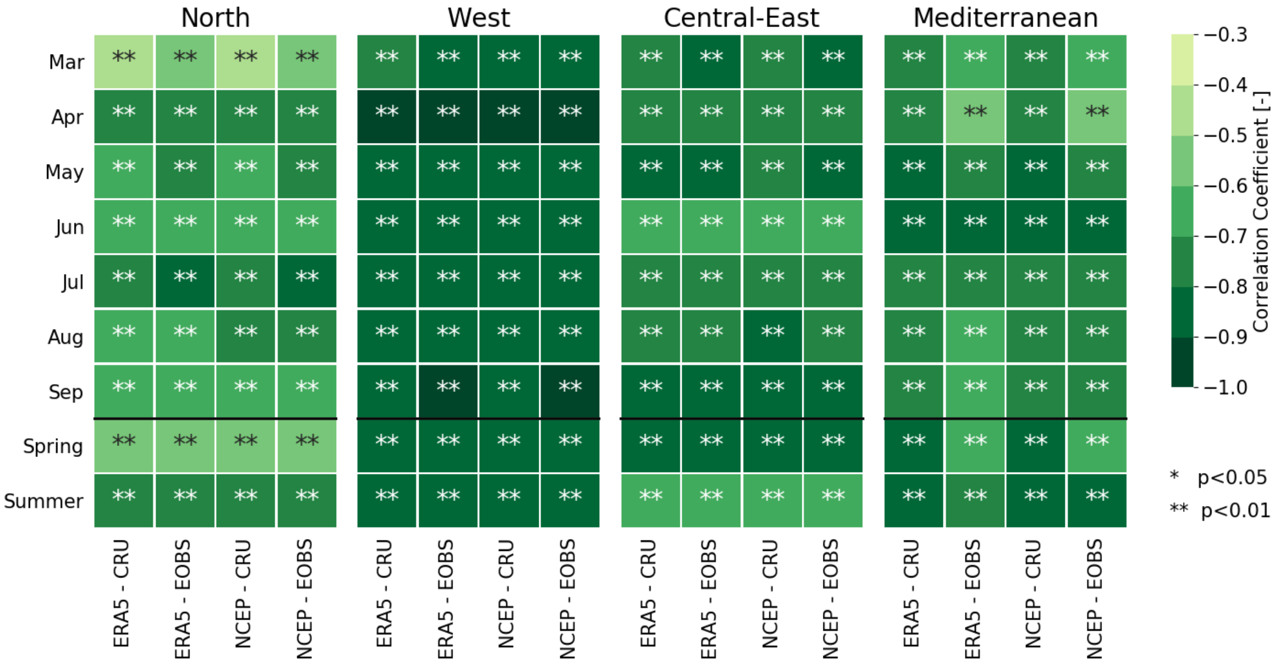

Figure S7: Rank correlation between regional Z500 and SPEI over the period 1979–2021 for the months March–September, as well as spring and summer. The rank correlations are calculated for each pair of Z500 (ERA5 or NCEP) and SPEI (CRU or EOBS) data sets. One star and two stars represent significant correlation at 5% level and 1% level, respectively.

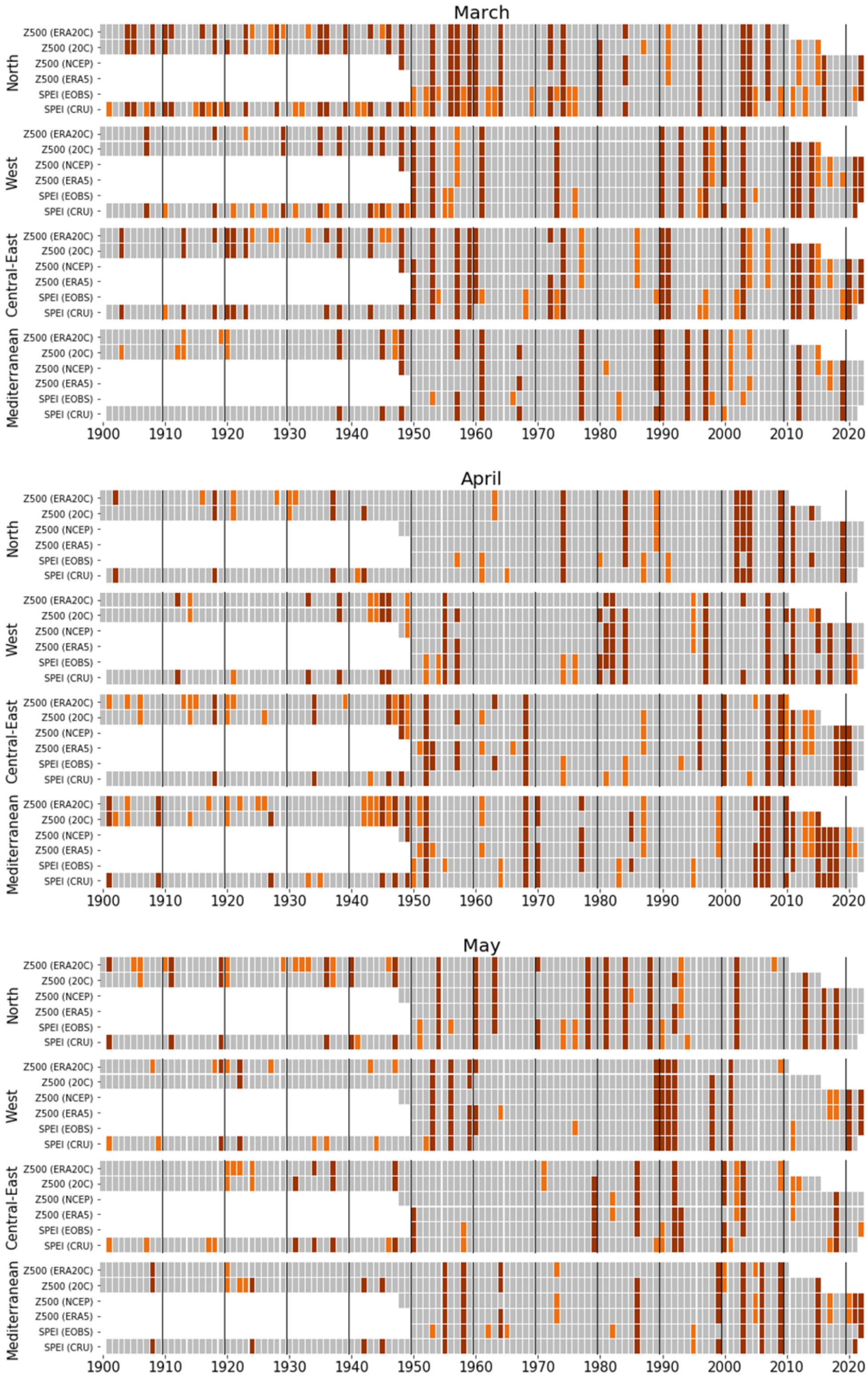

Figure S8: Regional 1900–2022 time series of anomaly occurrences for March, April and May. Anomalies are coloured dark red if at least one Z500 and one SPEI are co-occurring, and orange otherwise. Anomaly occurrences are defined as years of Z500>80th percentile (high-pressure anomaly), and years of SPEI<20th percentile (meteorological drought). Percentiles are computed from the reference period 1981–2010. Regions are defined as given by Fig. S5.

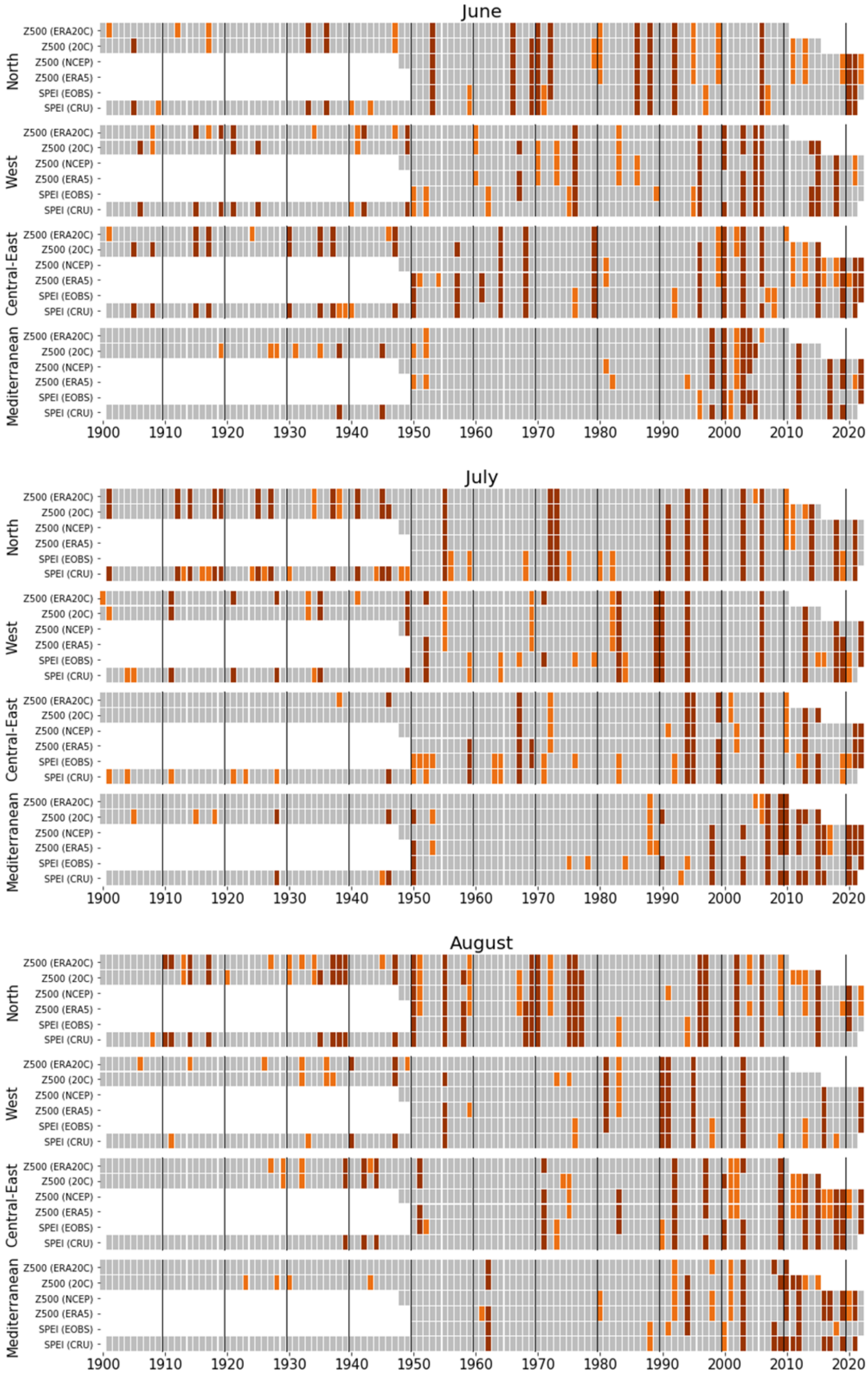

Figure S9: Same as Fig. S8, but for June, July and August.

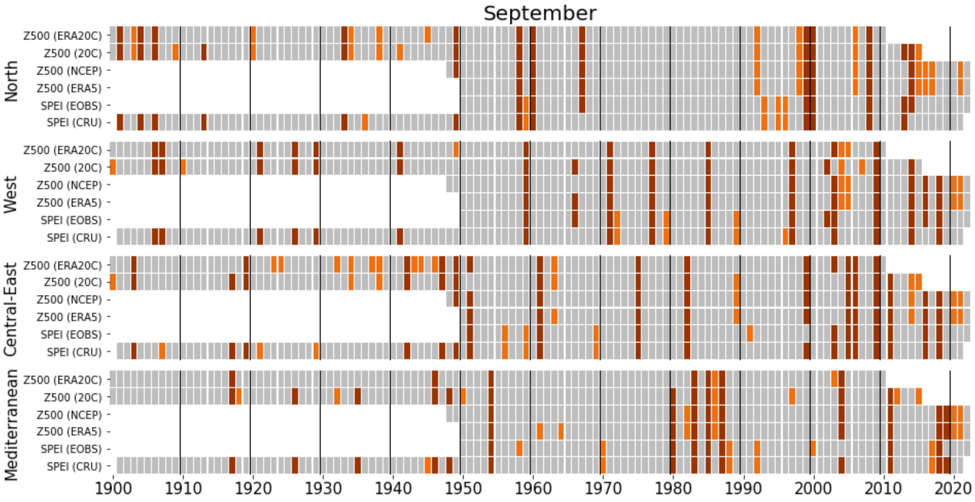

Figure S10: Same as Fig. S8, but for September.

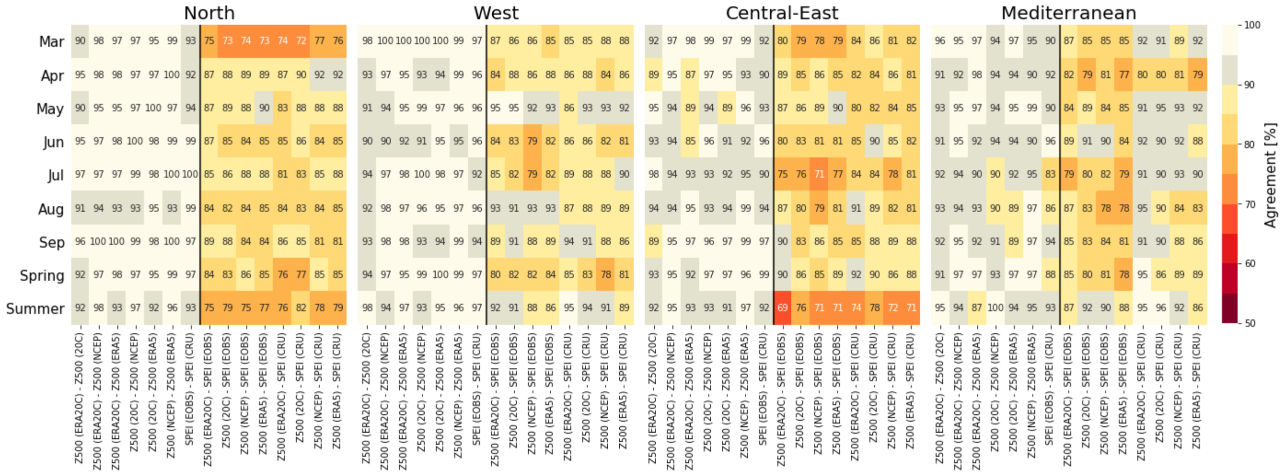

Figure S11: Percentage overlap between the anomaly occurrence time series (shown in Fig. 4 in the paper, and Supplementary Fig. S8–S10) as calculated for each pair of time series based on their shared period. Agreements between time series of the same variable are shown to the left and co-occurrence of Z500 and SPEI anomaly occurrence time series to the right of the black line.

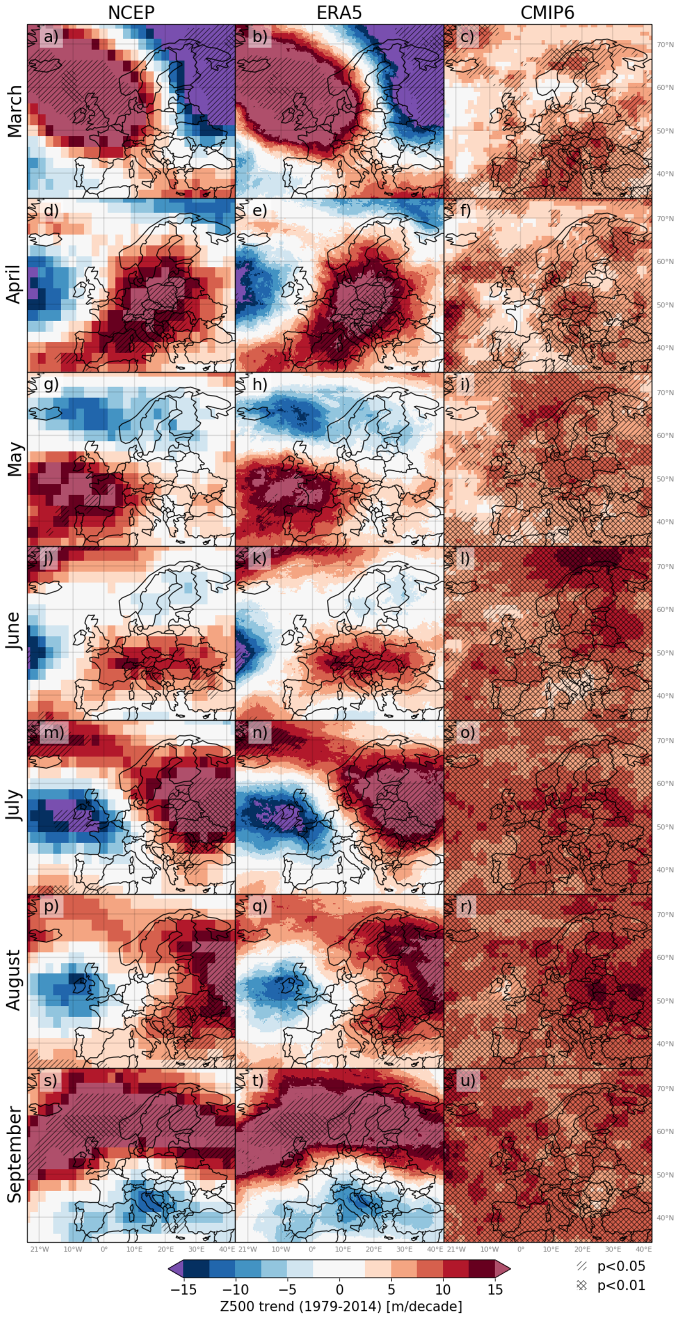

Figure S12: Gridwise Z500 trends in each month March (top panel, a–c) to September (bottom panel, s–u) over the period 1979–2014, using the data sets NCEP (left), ERA5 (middle) and CMIP6-Historical (right). Single-hatching and double-hatching represent areas with statistically significant trends at 5% and 1% level, respectively. The figure was created in Python v3.7.3 (`www.python.org`), using the package Cartopy v0.17.0 (`www.scitools.org.uk/cartopy/docs/v0.17/`).

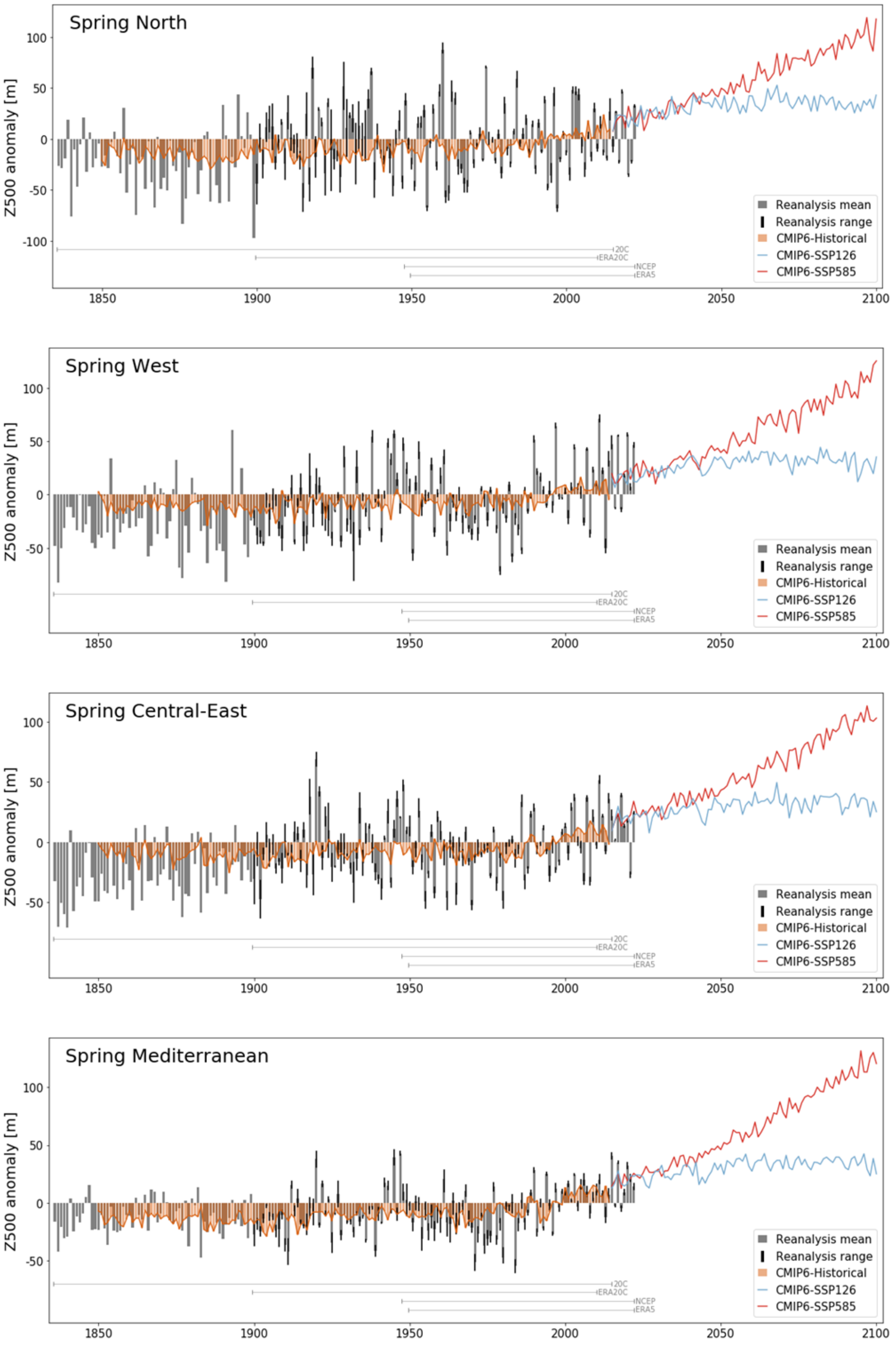

Figure S13: Spring Z500 anomalies (relative to 1981–2010) for the four regions (from top to bottom) North, West, Central-East and Mediterranean. Shown are mean (grey bars) and range (black lines) of the reanalysis data sets existing for the given year, and CMIP6-Historical (orange), CMIP6-SSP126 (blue) and CMIP6-SSP585 (red). Horizontal lines represent the reanalysis data sets' period coverage. Regions are defined as given by Fig. S5.

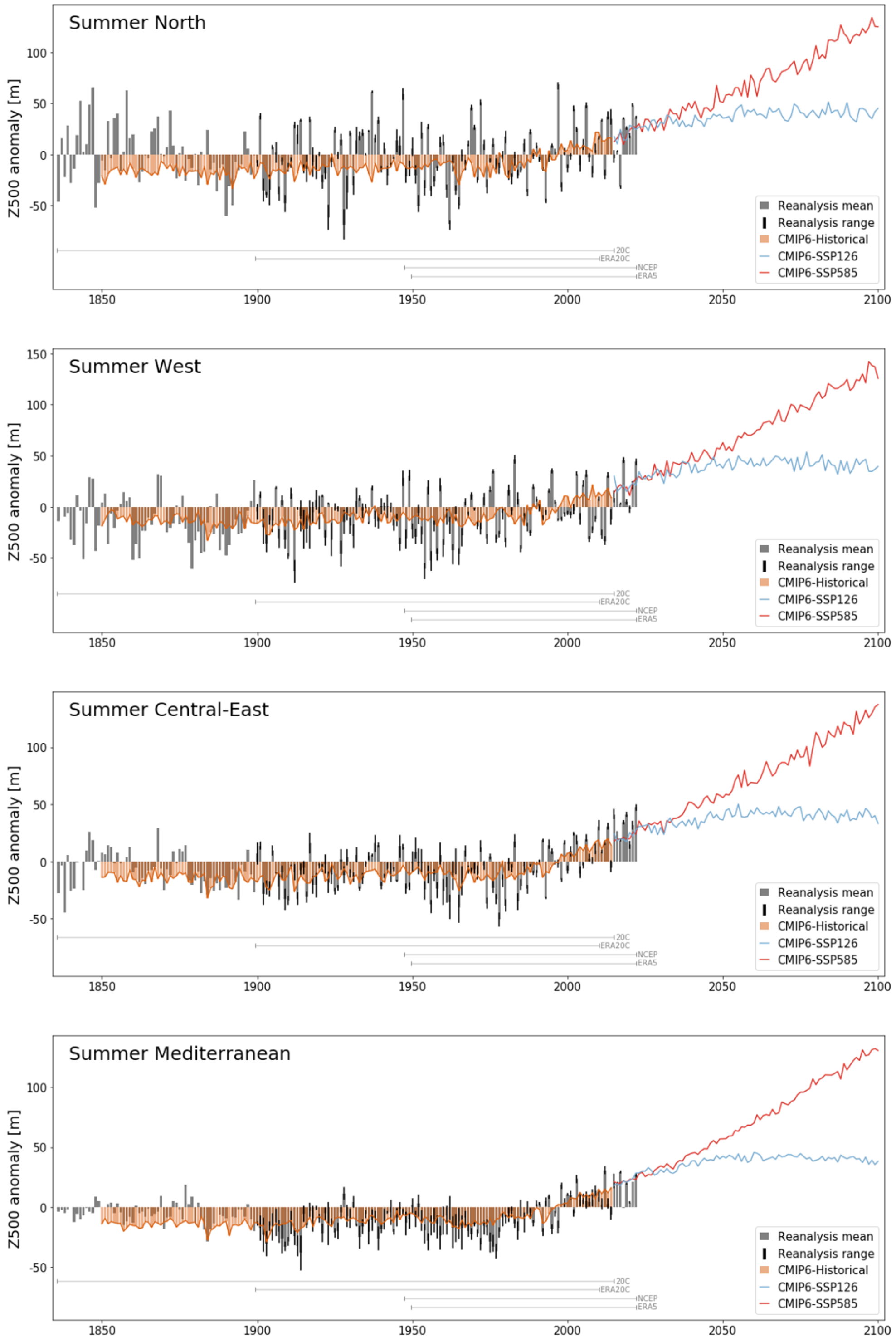

Figure S14: Same as Fig. S13, but for summer.

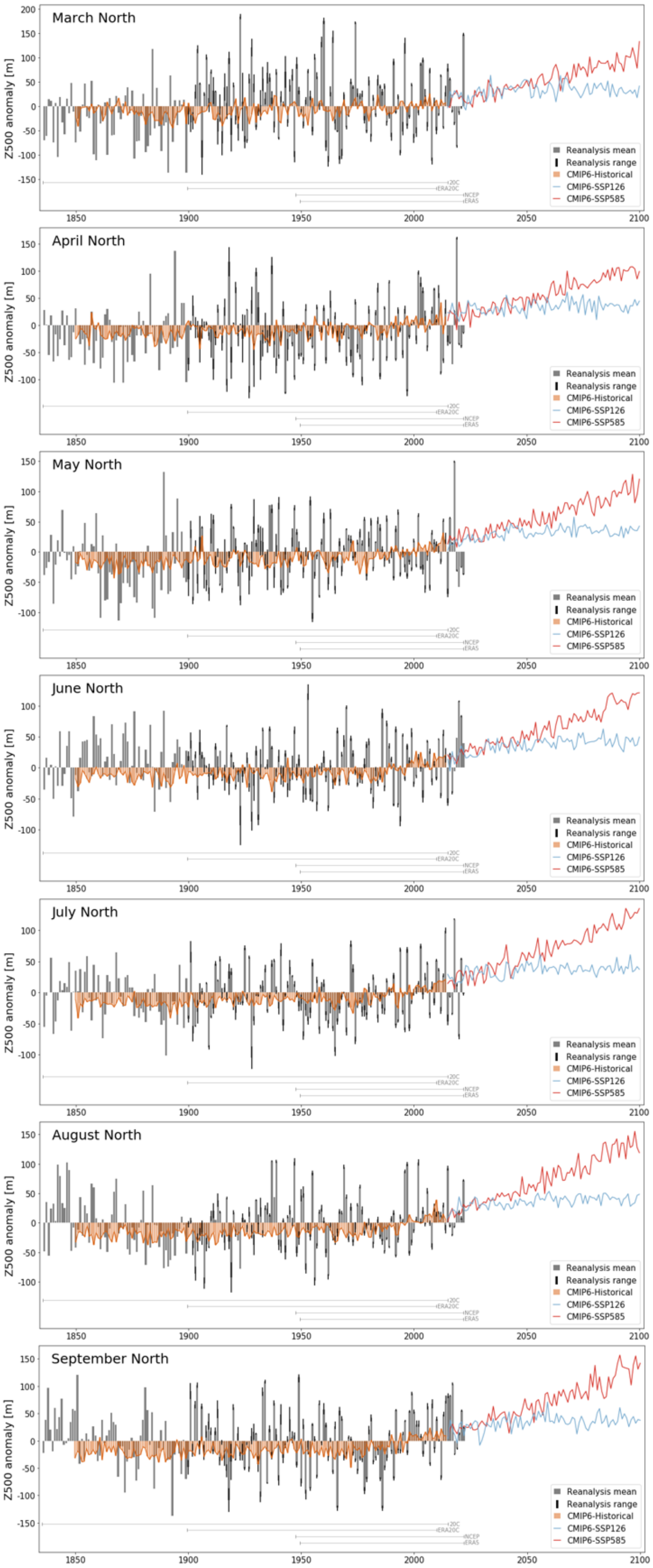

Figure S15: Monthly March–September Z500 anomalies (relative to 1981–2010) for the North region. Shown are mean (grey bars) and range (black lines) of the reanalysis data sets existing for the given year, and CMIP6-Historical (orange), CMIP6-SSP126 (blue) and CMIP6-SSP585 (red). Horizontal lines represent the reanalysis data sets' period coverage. The region is defined as given by Fig. S5.

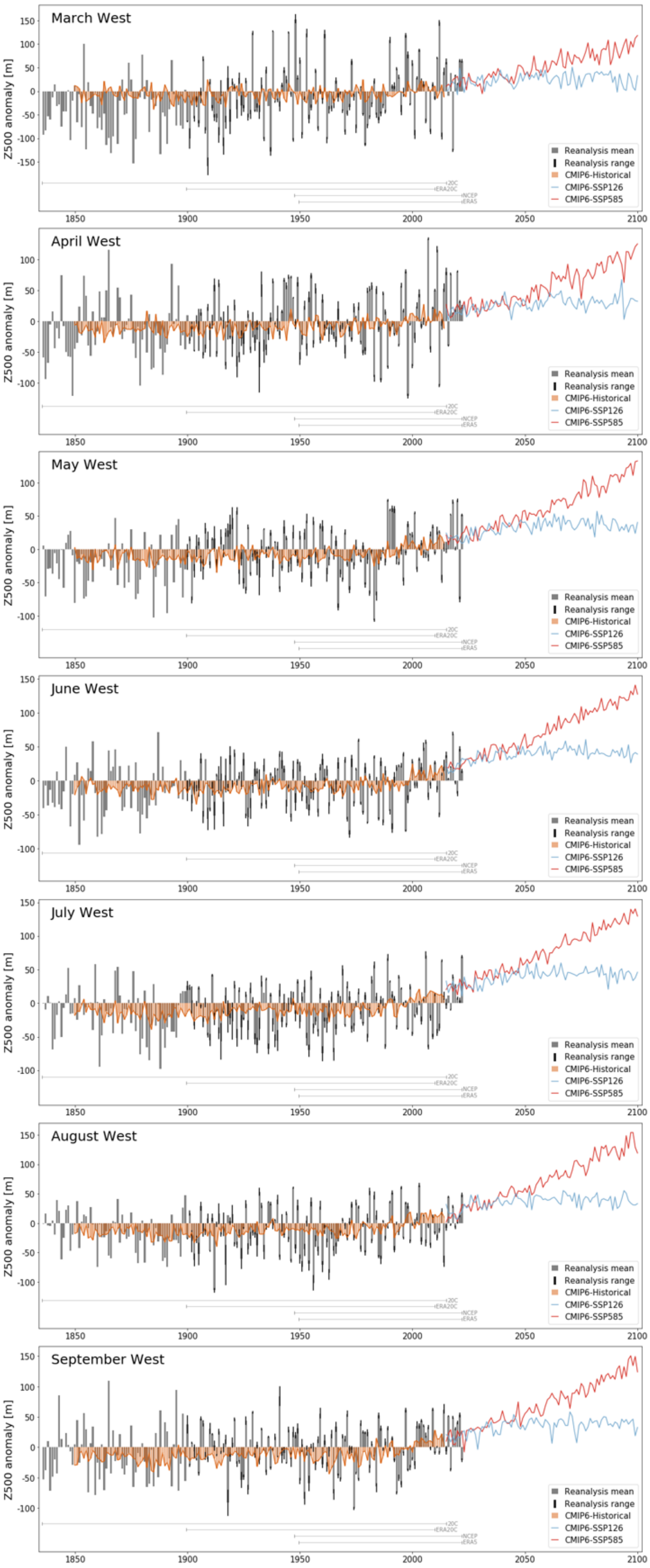

Figure S16: Same as Fig. S15, but for the West region.

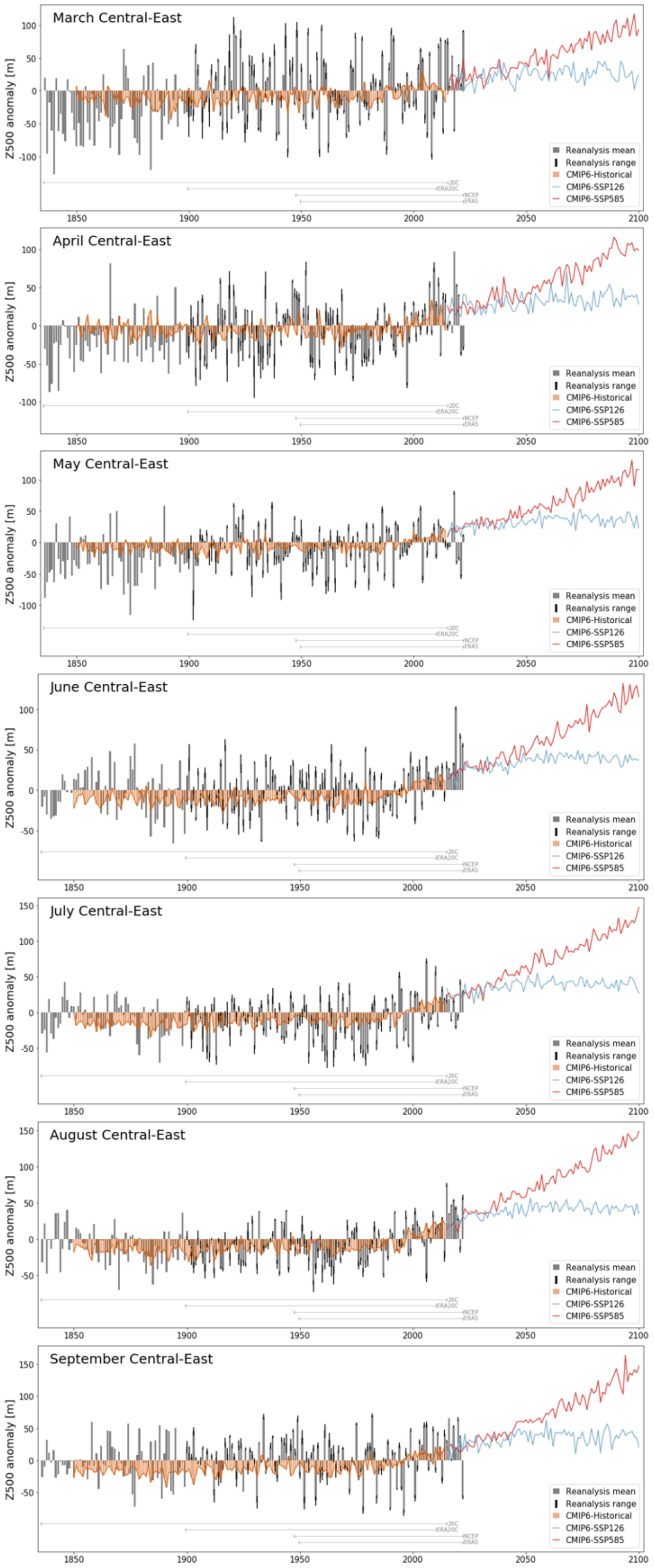

Figure S17: Same as Fig. S15, but for the Central-East region.

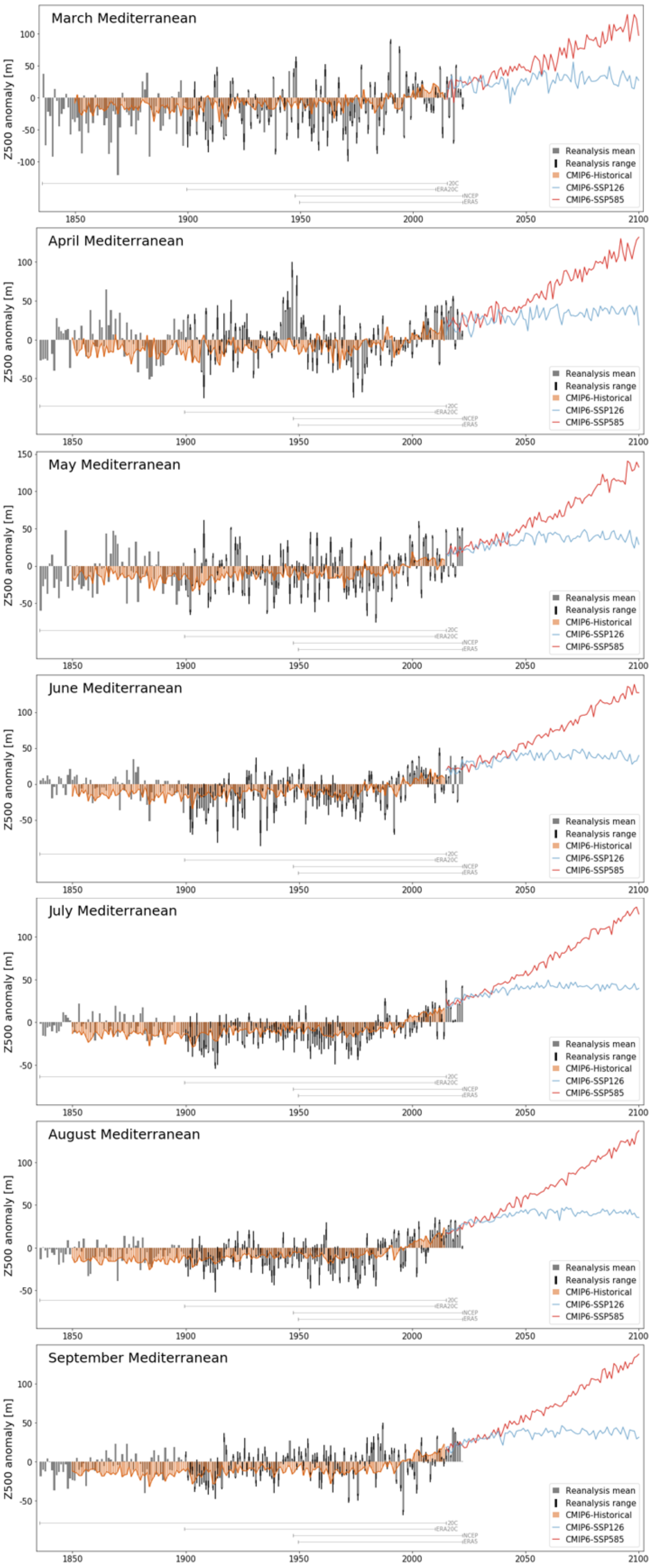

Figure S18: Same as Fig. S15, but for the Mediterranean region.

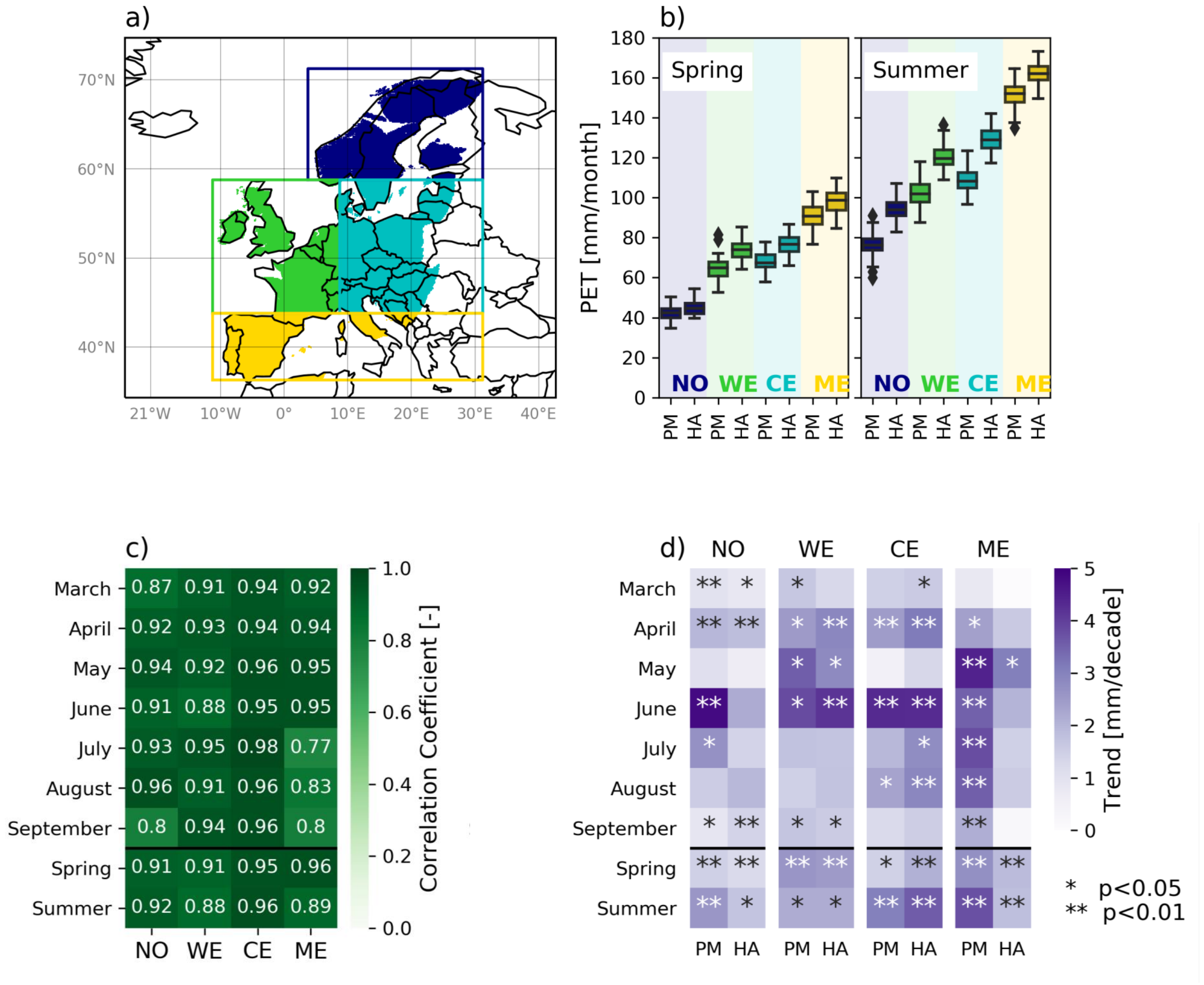

Figure S19: Comparison of E-OBS based Potential evapotranspiration (PET) by the Penman-Monteith method (PM) and the Hargreaves method (HA) over the shared period 1981–2020: a) Shared spatial coverage over March–September coloured according to climate regions as defined in Fig. S5; b) distributions (box plots) of the regional PET for spring and summer; c) Pearson correlation between regional PET by PM and by HA; d) Mann-Kendall trends of regional PET by PM and HA. The figure was created in Python v3.7.3 (`www.python.org`), using the package Cartopy v0.17.0 (`www.scitools.org.uk/cartopy/docs/v0.17/`).
